# Generative AI-based approach for player behavior analysis and gray area identification

**DOI:** 10.3389/frai.2026.1730018

**Published:** 2026-03-20

**Authors:** Vinay K., Sriram Sankaran

**Affiliations:** Center of Cyber Security Systems and Networks, Amrita Vishwa Vidyapeetham, Amritapuri, India

**Keywords:** bot detection, explainable AI (SHAP, LIME), generative AI (VAEs, GANs, CTGAN), gray-area behaviors, human-in-the-loop moderation, online gaming ecosystems, player behavior analysis

## Abstract

**Background:**

Detecting exploitative or unethical player behavior in online gaming platforms is challenging due to ambiguous gray-area actions that are neither clearly legitimate nor illegal.

**Methods:**

This study presents an interpretable behavior analysis framework combining anomaly detection, synthetic data augmentation using Conditional Tabular GAN (CTGAN), and ensemble learning with *post-hoc* explainability. Datasets from a massively multiplayer online role-playing game (MMORPG) spanning 88 days (49,739 player sessions) were augmented to address class imbalance. Anomaly detection using an Encoder-Decoder GAN for Anomaly Detection (EGBAD) approach generated anomaly-aware features. A stacked ensemble model combining Random Forest, XGBoost, and Artificial Neural Networks was developed, with SHAP and LIME providing explanations for predictions.

**Results:**

The proposed framework achieved 95.98% accuracy, 0.915 ROC-AUC, and 0.90 macro F1-score, outperforming baseline models. The integration of CTGAN improved minority class recall by 5–7 percentage points, while EGBAD-derived anomaly features enhanced gray-area detection. Human-in-the-loop triage for low-confidence predictions (6.8% of cases) achieved 75% human-AI agreement with reduced false positives (21% decrease) and false negatives (17% decrease).

**Discussion:**

The framework successfully balances automated detection with human oversight, providing transparent, interpretable decisions for player behavior moderation while maintaining fairness and reducing wrongful enforcement actions.

## 1 Introduction

Multiplayer online games have evolved into vast digital ecosystems, where millions of users participate in real-time interactions that include play, competition, and cooperation. Player behavior within these environments has significant implications for game balance, fairness, and community trust. The emergence of new user-generated content platforms, such as Roblox and Minecraft, has introduced additional complexity to these digital realms and the behaviors exhibited by players (Achiam et al., 2023; Ali et al., 2024). In contrast, player interactions in single-player game environments are more manageable and predictable. However, online games are characterized by continuous shifts and changes, presenting novel mechanics, emergent strategies, and evolving play strategies that are in constant flux. This perpetual change complicates the design of equitable enforcement systems, as disruptive behavior often manifests in subtle and adaptive forms (Afonso et al., 2024).

### 1.1 The pervasive problem: bots and gray-area behaviors

One of the most significant challenges in online gaming is the use of automated programs, called bots. These bots perform repetitive tasks, such as farming, trading, or leveling, which distort economies, inflate leaderboards, and degrade user experience. Their sophistication allows them to mimic human activities, complicating detection by anti-cheat mechanisms (Anzer and Stöcker, 2020; Baek et al., 2024). Equally concerning are behaviors in uncertain domains, such as the metaverse. These include activities that, while not directly violating game rules, exploit loopholes through macros, latency manipulation or semi-automated scripts. Such behaviors blur the lines between genuine and predatory play, providing unfair advantages and eroding trust in the gambling environment (Barnard et al., 2022; Bernardi et al., 2017). Beyond cheating, problematic play patterns linked to Internet Gaming Disorder (IGD) have raised global concerns among researchers and practitioners. Traditional approaches using self-report surveys are biased and limited (Bernardi et al., 2018), highlighting the need for telemetry-based behavior modeling to ensure fairness and digital wellbeing.

### 1.2 Challenges in current detection approaches

Current detection systems face several challenges in this regard.

- **Dynamic and evolving behavior**—Bots change their strategies quickly, so systems need to be able to adapt to new, unexpected strategies (Colledanchise and Ögren, 2018).
- **Class imbalance**—Since malicious accounts only make up a small part of logs, classifiers tend to lean toward normal activity (Cowley and Charles, 2016).
- **Ambiguity of gray-area actions**—Too strict of thresholds could lead to false positives, while too lax of thresholds could let people take advantage of the system (Chung et al., 2015).
- **Lack of explainability**—Moderators discover it more challenging to explain their behavior when machine learning models are insufficient. This can be problematic for players (Dai et al., 2024).

### 1.3 Motivation

Although anti-cheat technologies have advanced, an optimal balance between accuracy, adaptability, and transparency remains elusive. Rule-based detection systems are straightforward but are exploited as attackers adapt (Kannan and Sankaran, 2025; Achiam et al., 2023; Ali et al., 2024). Machine learning models enhance detection but function as opaque systems (Afonso et al., 2024; Anzer and Stöcker, 2020). This lack of transparency undermines trust when innocent players are misidentified by the system. Models struggle with class imbalances in game logs, where anomalous behaviors are rare (Baek et al., 2024). Classifiers become biased toward the majority class, reducing their effectiveness in identifying malicious activities. Gray-area activities such as macro-assisted playing remain inadequately addressed (Barnard et al., 2022; Bernardi et al., 2017).

To maintain fairness in online gaming, detection frameworks must combine accuracy and interpretable outputs. Generative AI can address data imbalance by modeling behavioral distributions and generating minority-class samples (Bernardi et al., 2018; Colledanchise and Ögren, 2018). Integration with XAI tools enhances transparency for enforcement decisions (Cowley and Charles, 2016). A human-in-the-loop system ensures that ambiguous cases are reviewed, reducing false positives while maintaining efficiency (Chung et al., 2015; Dai et al., 2024). This study aims to bridge the gap between detection performance and accountability, thereby advancing trustworthy solutions for monitoring online player behavior.

### 1.4 Contributions of this study

This study presents advances in player behavior analysis and bot detection through a Generative AI system that combines anomaly detection, synthetic data, ensemble learning, explainability, and human supervision. The key contributions of this study are as follows:

1. **Player behavior using generative models**—VAEs and GANs reveal hidden player behavior, enabling detection of obvious bots and soft exploits.
2. **Anomaly-aware features**—EGBAD integration enables autoencoder reconstruction errors as indicators of gameplay deviation.
3. **Synthetic minority augmentation**—CTGANs resolve unbalanced game logs by generating minority class samples, improving classifier recall.
4. **Ensemble learning architecture**—Stacked ensembles of Random Forests, XGBoost, and Neural Networks achieve 95.9% accuracy.
5. **Explainable AI integration**—SHAP and LIME help moderators make transparent decisions and provide local and global prediction understanding.
6. **Human-in-the-loop triage**—Gray-zone filtering sends low-confidence predictions to human reviewers for contextual judgment.

These contributions create a unified framework for advancing fair and interpretable solutions for online gaming ecosystems.

### 1.5 Paper organization

This paper is organized as follows: Section 2 reviews the literature on player behavior modeling, bot detection, generative AI applications in gaming, and explainable human-in-the-loop frameworks. Section 3 introduces the proposed generative AI-based framework, elaborating on its pipeline components, which include anomaly aware feature extraction, conditional generative adversarial network (CTGAN)-based data augmentation, ensemble modeling, explainability, and human-in-the-loop modules. Section 4 outlines the experimental setup, including the dataset, preprocessing methods, baseline models, evaluation metrics and computational environment. Section 5 presents the results and discussion, encompassing the model performance, impact of augmentation, analyses of explainability, and key findings and discussion. Finally, the Conclusion Section summarizes the paper's contributions and suggests future research directions.

## 2 Related work

Player behavior analysis and anomaly detection in games span multiple domains, including player modeling, bot detection, generative AI, behavioral health, and human-centered explainability. This section reviews prior research in these strands to situate the proposed framework for the study.

### 2.1 Player behavior modeling and profiling

Player behavior modeling has been pivotal in game analytics, focusing on how players engage with complex digital environments. Initial investigations used basic telemetry analysis, deriving metrics such as session duration and win/loss ratios to categorize player types (Kannan and Sankaran, 2025; Achiam et al., 2023; Ali et al., 2024). These aggregate measures offer limited insights into gameplay patterns. The Mechanics-Dynamics-Aesthetics framework conceptualizes games as systems in which mechanics drive actions, dynamics emerge from interactions, and aesthetics capture experiences (Afonso et al., 2024). The Behavlets framework integrates psychological traits with telemetry features to create descriptors of action sequences (Anzer and Stöcker, 2020; Mahlmann et al., 2010; Zouhri et al., 2025). While these approaches are valuable in combining cognitive and behavioral perspectives, their reliance on handcrafted features limits their scalability (Smirnov et al., 2024).

Research has explored data-driven profiles through sequence mining and clustering of telemetry logs to identify recurrent play motifs (Baek et al., 2024). Object-oriented models encode rules to simulate outcomes (Bernardi et al., 2017; Mahlmann et al., 2010; Zouhri et al., 2025), while game-theoretic approaches assume rational agents to predict actions (Barnard et al., 2022). Challenges persist as models remain genre-specific, working well in MMORPGs but less so in sandbox games (Bernardi et al., 2018). Traditional profiling methods struggle in dynamic environments where strategies evolve, limiting the real-time detection of disruptive activities. While prior research has established foundations for understanding player behavior, existing models lack scalability and adaptability, presenting opportunities for generative approaches that capture behavioral structures while providing interpretable insights (Colledanchise and Ögren, 2018; Zouhri et al., 2025; Smirnov et al., 2024).

### 2.2 Bot detection and gray-area identification

The integrity of fairness and trust within online multiplayer game communities is at risk because of destabilizing factors. Bot infiltration remains a major challenge, as they provide users with unfair advantages by performing tasks such as farming and resource gathering. Early detection attempts used machine-learning classifiers such as KNN, SVM, and decision trees (Achiam et al., 2023), along with behavioral features such as action frequency and session length distribution (Ali et al., 2024). Although these approaches have been successful in some cases, they struggle to adapt to new tactics (Kannan and Sankaran, 2025; Nedungadi et al., 2025).

Ethical frameworks for player modeling have been explored (Mikkelsen et al., 2017), though Random Forests and hybrid models combining neural networks with tree-based classifiers (Afonso et al., 2024; Anzer and Stöcker, 2020). However, these methods rely on manual features and thresholds, which imposters can exploit by mimicking human patterns (Baek et al., 2024). Gray-area behaviors like semi-automated macros and latency exploitation make distinguishing between cheating and legitimate play difficult, raising ethical concerns despite not explicitly violating game rules (Jithish et al., 2024).

This challenge is complicated by class imbalance in gray-area detection, as malicious players comprise a small fraction of users. This imbalance makes classifiers less sensitive to uncommon but disruptive behaviors. Adaptive frameworks using anomaly detection with human intervention have gained attention for distinguishing between bots, gray-area behaviors, and real players. While bot detection has progressed from statistical heuristics to ensemble frameworks, gray-area behaviors require models that emphasize precision and adaptive learning (Nedungadi et al., 2025).

### 2.3 Generative AI and large language models in games

The rise of generative artificial intelligence (AI) has facilitated player behavior analysis. Unlike traditional discriminative models, generative models recover gameplay data distribution, allowing them to uncover gradual behavioral changes that might go unnoticed by users and indicate game disruption.

Major representatives of player telemetry include Variational Autoencoders (VAEs) and Generative Adversarial Networks (GANs). VAEs convert behavioral logs into dense latent representations, facilitating anomaly detection using reconstruction errors (Achiam et al., 2023; Mnih et al., 2015). GANs, particularly those with an encoder-decoder structure, isolate abnormal gameplay and generate realistic player trajectories for training (Ali et al., 2024; Mao et al., 2024). CTGAN is a GAN subclass that reuses machine learning tasks with imbalanced datasets to produce synthetic samples while preserving feature correlation to improve classifier performance on rare behaviors (Afonso et al., 2024). Large Language Models (LLMs) have transformed game scenarios through reasoning and content generation, initially generating quests, narratives, and levels that adapt to player actions (Anzer and Stöcker, 2020; Baek et al., 2024; Mao et al., 2024). Systems such as Voyager demonstrate LLMs as self-sufficient agents that create strategies and gameplay decisions (Barnard et al., 2022; Ma et al., 2024).

In hybrid methods, LLMs design policies and behavior trees for lower-level agent execution (Bernardi et al., 2017; Mao et al., 2024). This approach reduces the computational costs while maintaining interpretability. Challenges include grounding LLM reasoning with flight data, achieving game-independent generalization, and reducing output volatility (Bernardi et al., 2018; Colledanchise and Ögren, 2018; Ma et al., 2024). Generative AI serves as a multimodal toolset for anomaly detection, data augmentation, and adaptive content generation; however, its implementation with explanations and human supervision remains an active research area.

### 2.4 Recognition of problematic gaming behaviors

Investigators' attention has shifted from broader gaming to in-game long plays and Internet Gaming Disorder (IGD), including cheating and exploitation. The authors note that IGD, as per the DSM-5, requires additional research, raising questions about digital well-being and health risks from online over-involvement (Achiam et al., 2023).

IGD symptom identification previously relied on self-report questionnaires and clinical interviews, which provided a psychological context but faced scalability issues and bias (Ali et al., 2024). Recent studies have explored sensor-equipped behavior tracking, measuring session duration, playtime, and login frequency to detect excessive gaming (Afonso et al., 2024; Anzer and Stöcker, 2020; Mnih et al., 2015). Researchers have integrated game activity records with facial recognition and voice-based emotion analysis to detect stress, frustration, and compulsive play symptoms (Baek et al., 2024).

However, limitations remain. Collecting sensitive game parameters and biometric data raises privacy concerns (Bernardi et al., 2017), while cultural differences complicate the establishment of universal thresholds for problematic play (Bernardi et al., 2018). Anti-cheat frameworks remain incompatible with IGD-focused approaches that separate problematic gaming from exploitative behaviors. These gaps demonstrate the need for integrated frameworks that support fairness and digital well-being in online gaming.

### 2.5 Human-in-the-loop and explainable AI approaches

Explainability and human-in-the-loop are becoming crucial as machine learning models are used in high-stakes digital ecosystems to ensure their accountability and reliability. In online gaming, moderators need interpretable proof of the reasons behind flagging suspicious player behavior.

Explainable AI (XAI) methods, including SHapley Additive exPlanations (SHAP) and Local Interpretable Model-Agnostic Explanations (LIME), help attribute model predictions to input features (Achiam et al., 2023). SHAP values quantify feature contributions to predictions, whereas LIME generates local approximations of complex models (Ali et al., 2024). While effective in cybersecurity and healthcare, these methods remain nascent in gaming analytics (Afonso et al., 2024; Mitterhofer et al., 2009). Human-in-the-loop (HITL) systems bridge automated detection and human judgement by forwarding low-confidence predictions to human reviewers for validation (Anzer and Stöcker, 2020). This approach combines automation efficiency with human context (Baek et al., 2024; Mitterhofer et al., 2009).

Interactive visualization dashboards combine anomaly scores with explanatory insights for moderator assessment (Barnard et al., 2022). These systems enhance transparency and reduce disputes arising from wrongful bans. However, research has typically treated explainability, anomaly detection, and HITL as separate components (Bernardi et al., 2017; Mitterhofer et al., 2009; Mnih et al., 2015). This highlights the need for frameworks that integrate generative modeling, explainability, and human oversight to balance accuracy and fairness, ensuring transparent and accountable enforcement decisions in gaming communities (Sunku Mohan et al., 2025).

Table 1 presents prior studies on player behavior analysis and enumerates the player analysis extracted for behavioral modeling. These features span temporal signals (e.g. action timestamps and response latencies), spatial traces (movement vectors and pathing loops), micro-level gameplay actions (crafting sequences and item transactions), and multimodal cues (chat patterns and facial micro-expressions). Each feature provides an interpretable behavioral signal that helps distinguish between legitimate human play and automated or gray-area behaviors. For instance, action timing irregularities or jittered trajectories often indicate human variability, whereas rigid periodicity or deterministic loops are hallmarks of robotic behaviors. Affective outbursts and micro-social cues can also reveal problematic gaming or a lack of genuine engagement in scripted clients. This enriches the foundation for anomaly detection, explainability, and human-in-the-loop moderation in intricate gaming ecosystems (Kang et al., 2016; Kotkov, 2018).

**Table 1:** Categorization of micro-level gameplay attributes derived from player telemetry, including frequency-based, temporal, and sequence-driven behavioral indicators used for bot and gray-area behavior detection.

**Feature (type)**	**Description**	**Why it matters**	**Extraction method**	**Citation**
Action timestamp sequence (temporal)	Millisecond/second-level timestamps of atomic actions (move, attack, trade, collect)	Reveals rhythm, inter-action intervals, robotic periodicity, session micro-patterns	Event log parsing → inter-event intervals, burst detection, interquartile ranges	Bernardi et al., 2017; Chung et al., 2015
Per-action latency/response time (temporal)	Time between stimulus and player response (e.g., target acquisition to fire)	Distinguishes human reaction variability from deterministic bot timing	High-resolution trace required; rolling statistics, autocorrelation	Drachen et al., 2009
Movement vectors	Fine spatial coordinates/trajectory segments within a session	Captures pathing regularity, teleport-like jumps, low-variance farming loops	Trajectory segmentation, path similarity metrics (Frechet, DTW), entropy measures	Cowley and Charles, 2016; Guérin et al., 2017; Holmgård et al., 2014
Click/command histogram (micro-frequency)	Counts of specific action types in short windows (e.g., 1s, 5s)	Exposes rapid repetitive behavior and mechanically precise loops	Sliding-window counts, Poisson tests	Barnard et al., 2022; Galway et al., 2009; Giacomello et al., 2018
Micro-resource transactions (trade/item events)	Time-stamped item/trade events with quantities	Highlights automated farming or scripted trading patterns	Event cross-correlation, sequence motifs, Markov transition counts	Dai et al., 2024; Kang et al., 2023
Per-item use sequences (stateful)	Ordered sequences of inventory use/craft actions	Detects deterministic crafting loops vs. adaptive human choices	Sequence mining, n-gram / sequential pattern mining, edit distance	Bernardi et al., 2018; Houlette and Rabin, 2004
Micro-social interactions (chat, whispers, emojis)	Short messaging patterns and timing	Bots often have absent/sterile social signals or templated replies	NLP template detection, reply latency, lexical diversity metrics	Drachen et al., 2012; Gallotta et al., 2024; Irfan et al., 2019
Sensor / client telemetry spikes (system-level micro)	Packet timing jitter, repeated network calls	Can indicate headless clients or scripted network behavior	Network trace analysis, clustering of telemetry fingerprints	Gramelt et al., 2024; Kotla, 2023
Facial micro-expressions/affect bursts (multimodal)	Short emotion spikes during play (if available)	Augments behavioral signal for problematic gaming and engagement	Frame-level affect detection (e.g., DeepFace), alignment to events	Ali et al., 2024; Kotkov, 2018

### 2.6 Research gaps and novelty

From traditional statistical profiling (Achiam et al., 2023; Ali et al., 2024) to ensemble learning for bot detection, generative AI for anomaly modeling (Baek et al., 2024; Barnard et al., 2022), and frameworks for problematic gaming (Bernardi et al., 2017, 2018), literature shows advances in player behavior analysis. Developments in explainable AI (XAI) and human-in-the-loop systems show potential for equitable decision-making (Colledanchise and Ögren, 2018; Cowley and Charles, 2016).

Despite these advances, there are still significant gaps.

- Approach Fragmentation- Studies treat detection, explainability, and human oversight separately rather than as an integrated pipeline, resulting in systems either accurate but opaque or interpretable but limited.
- Understudied gray-area behaviors- While bot detection receives attention, behaviors like semi-automated macros remain poorly understood due to overlap with legitimate play (Chung et al. 2015).
- Data Imbalance- Frameworks are limited by class disparity in rare disruptive behaviors. Explainability and ensemble learning lack integration, despite CTGAN methods (Dai et al. 2024).
- Ethical Issues- Privacy, fairness, and cultural context rarely translate into practical frameworks balancing automation and oversight (Drachen et al. 2009).

These gaps inform our study, presenting a unified generative AI framework that (i) uses anomaly aware features and CTGAN augmentation, (ii) incorporates ensemble models with explainability, and (iii) includes human-in-the-loop triage for gray-zone cases. This framework enhances player behavior analysis by addressing both ethical and technical challenges.

Earlier studies aggregating player behavior over longer time scales are summarized in Table 2. Unlike Table 1, which records millisecond-level actions, it shows scalable indicators such as playtime, session length, economic activity, and achievement progression. These traits help in identifying gaming disorders and monitoring long-term patterns. However, these techniques may overlook subtle exploitative patterns in short time windows. This emphasizes the need for a hybrid framework that integrates both fine- and coarse-grained signals (Kang et al., 2016).

**Table 2:** Grouping of session-level and aggregated behavioral metrics that show patterns of cooperation, economic activity, and general engagement throughout gaming sessions.

**Feature (type)**	**Description**	**Why it matters**	**Extraction method**	**Citation**
Total playtime (per day/week)	Aggregated time spent in sessions across intervals	Identifies excessive gaming, potential IGD, or bot-like 24/7 play patterns	Log aggregation, moving averages, z-score thresholds	Ali et al., 2024; Kang et al., 2023
Session length distribution	Average and variance of continuous play sessions	Long uninterrupted sessions may suggest bots or problematic play	Session segmentation from login/logout logs, histogram fitting	Chung et al., 2015; Houlette and Rabin, 2004
AI with Extreme Programming (XP)	Speed of advancement in experience points or levels	Abnormal acceleration indicates exploitation or automation	Curve fitting of XP progression vs. time, outlier detection	Barnard et al., 2022; Guérin et al., 2017; Holmgård et al., 2014
Economic activity aggregates	Total trades, gold earned, or resources collected per session/day	Detects farming behaviors, macro exploitation, or RMT activities	Summed transaction logs, ratios of inflow/outflow	Dai et al., 2024; Kotla, 2023
Social activity aggregates	Number of chat messages, party joins, guild participation per session	Lack of engagement may signal automation; over-engagement may flag spammers	Log counting, entropy measures, ratio of solo vs. social play	Drachen et al., 2012; Gallotta et al., 2024; Irfan et al., 2019
Achievement/quest completion rates	Rate of quest or badge completion	Bots may rush linear content abnormally fast; IGD players may grind repetitively	Aggregated quest/event logs, time-to-completion ratios	Bernardi et al., 2017; Drachen et al., 2009
Win/loss ratios or performance metrics	Aggregate success in matches, raids, PvP	Extreme imbalance may signal smurfing, boosting, or scripted play	Match logs, ELO/MMR analysis, distribution fitting	Bernardi et al., 2018; Galway et al., 2009; Giacomello et al., 2018
Cross-session behavioral stability	Consistency of play style across weeks/months	High stability = bot-like; adaptive variability = human	Rolling window similarity metrics, clustering stability scores	Cowley and Charles, 2016; Gramelt et al., 2024
Emotion/affect trends (macro-level)	Long-term averages of emotional states during gameplay	Persistent negative affect correlates with problematic gaming or burnout	Aggregated multimodal signals, time-series smoothing	Ali et al., 2024; Kotkov, 2018

## 3 Proposed framework

We propose a generative AI-based framework for analyzing player behavior and locating these gray areas to overcome the limitations of earlier studies. Unlike conventional detection systems that depend on isolated classifiers or static thresholds, our framework integrates XAI, generative data augmentation, ensemble learning, anomaly aware feature extraction, and human-in-the-loop triage into a single pipeline. This design ensures social accountability and technical robustness, thereby enabling scalable and reliable player monitoring in online gaming ecosystems. The framework is based on four guiding principles.

- Reproducibility—Every step of the pipeline, from preprocessing to modeling, is designed to be modular and replicable, which facilitates deployment in various gaming environments.
- Extensibility—The architecture is made to accommodate the integration of emerging AI architectures (such as transformers) and the addition of new modalities (such as voice data and chat logs).
- Scalability—The system's use of generative augmentation and ensemble learning allows it to handle large, unbalanced datasets without compromising performance.
- Governance and Accountability—The framework uses human-in-the-loop supervision and SHAP/LIME explanations to ensure transparency and equity in enforcement.

### 3.1 Overall pipeline architecture

**Figure 1:**
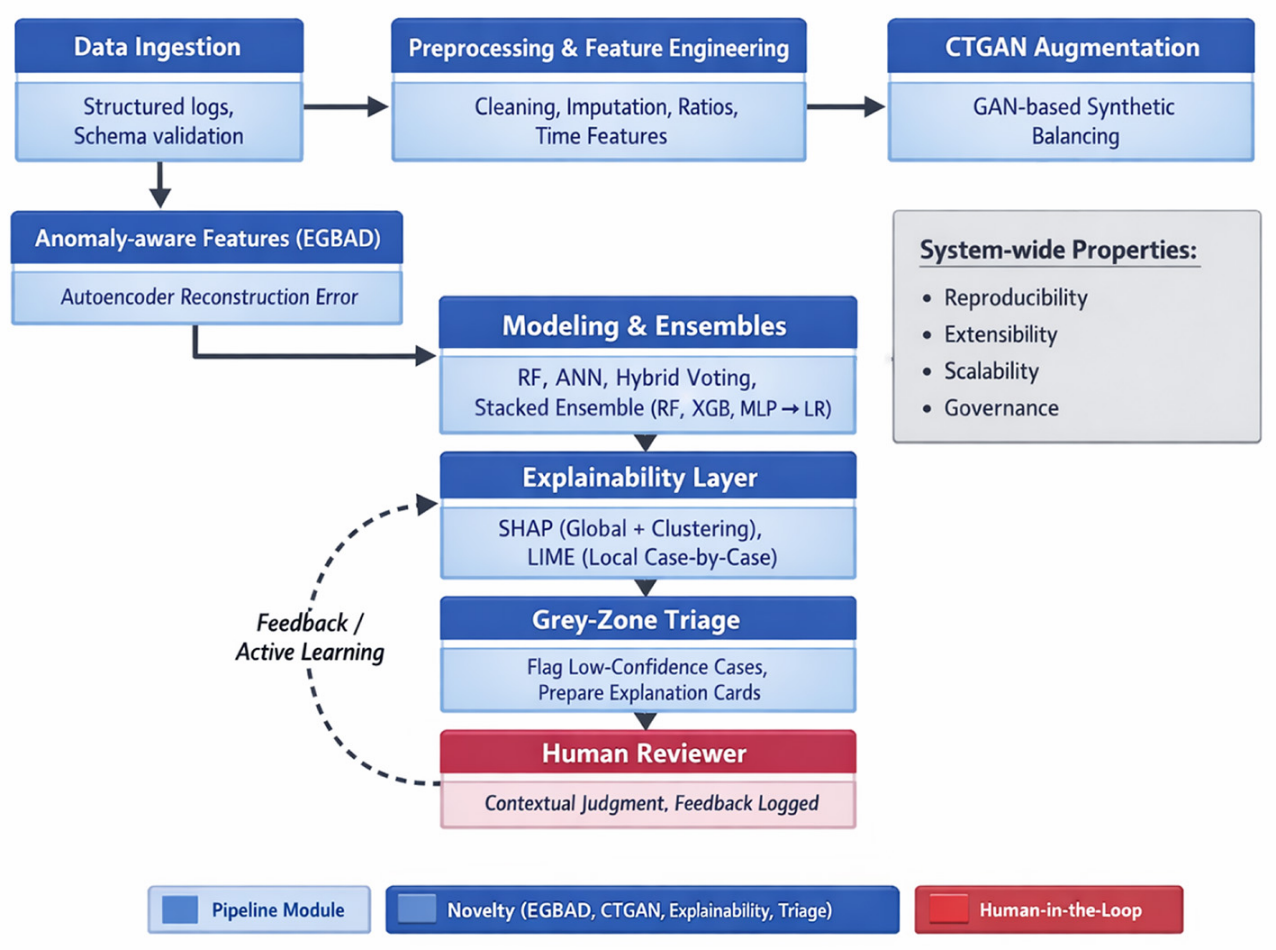
System architecture for player behavior analysis.

By integrating multiple components into a smooth end-to-end pipeline, the proposed structure effectively strikes a balance between interpretability, scalability, and accuracy. The modular design of the overall system architecture includes explainability, data ingestion, preprocessing, anomaly aware feature extraction, generative augmentation, ensemble modeling, and human-in-the-loop triage, as depicted in Figure 1. Similar modular architectures have been employed for fraud detection (Achiam et al., 2023) and cybersecurity (Ali et al., 2024) and gaming analytics (Afonso et al., 2024). However, our design uniquely incorporates generative AI and explainability to address the dual challenges of bot detection and gray-area identification (Chandran et al., 2025).

The pipeline begins with the ingestion of 49 K player data with 65 features. Schema validation and metadata cataloging ensure consistency across datasets (Anzer and Stöcker, 2020). The preprocessing stage involves sessionization, missing value imputation, and normalization. Coarse-grained aggregates and fine-grained features, such as inter-event intervals, form the basis of anomaly aware modeling (Baek et al., 2024). The encoder-decoder GAN anomaly detection estimates reconstruction errors and anomaly scores for player sessions (Barnard et al., 2022). CTGANs generate synthetic minority-class samples to address class imbalance (Bernardi et al., 2017). The datasets were combined to create a balanced input for the classifiers.

The framework includes an explainability module, where SHAP provides feature attribution and LIME generates local explanations (Colledanchise and Ögren, 2018). Insights are clustered using t-SNE and k-means, helping moderators to detect systematic anomalies (Cowley and Charles, 2016). Low-confidence predictions are directed to gray-zone triage, where reviewers access anomaly scores and explanations to evaluate cases (Chung et al., 2015; Shan and Michel, 2024).

The system enables online scoring for real-time detection and batch analysis. Models are containerised for scalable inference, while monitoring studies evaluate variants and guide active learning (Dai et al., 2024). This architecture improves player behavior analysis by combining explainability, generative augmentation, and human oversight.

### 3.2 Data ingestion and preprocessing

To ensure that later modules run on dependable, consistent, and instructive input, the proposed framework depends on sound data ingestion and preprocessing. A systematic approach to data preparation is required to facilitate accuracy and reproducibility because raw game telemetry data are highly susceptible to noise, variation, and significant gaps in data (Achiam et al., 2023; Ali et al., 2024).

#### 3.2.1 Data acquisition and structure validation

The ingestion process is responsible for taking in structured telemetry recorded during gameplay in a format which is generally either tabular (CSV, SQL exports) or log-structured (JSON). Data schema validation was used to confirm the presence and type of fields (timestamps, types of events, identifiers, and class labels) (Afonso et al., 2024). Summary statistics are calculated at this phase to examine distributional properties, outliers, and schema inconsistencies, as this will prevent unnoticed bias and errors from propagating failures (Anzer and Stöcker, 2020). Metadata catalogs also delineate schema versioning in support of longitudinal tracking and reproducibility across changing datasets (Baek et al., 2024).

#### 3.2.2 Data cleaning and normalization

After data acquisition, the data were transformed into a standardized feature matrix suitable for machine learning. Inconsistent records and duplicate entries were eliminated, and computational inference was used to manage missing values. Character class and event type were examples of categorical features that were subjected to mode imputation, whereas session length and total time played were examples of continuous variables that were subjected to median imputation (Barnard et al., 2022). This approach balances simplicity and robustness while maintaining the representativeness of the dataset.

#### 3.2.3 Field-specific feature engineering

In addition to standard preprocessing, the system emphasizes gaming-related feature engineering, transforming data into behavioral indicators that reveal refined patterns of play (Colledanchise and Ögren, 2018). The key categories included the following:

- Money per trade and experience per playtime are two examples of ratio features that demonstrate in-game efficiency.
- Item frequency features that indicate automated farming or macro use track repetitive event counts (item acquisitions per day, idle events, kill counts).
- Temporal features are time-aware aggregates that help identify irregularities in player engagement patterns. Examples include average session duration, circadian activity cycles, and inter-event intervals (Cowley and Charles, 2016).

These engineered features are especially helpful in separating genuine high-engagement players from bots or gray-area actors who repeatedly or unnaturally take advantage of game mechanics.

#### 3.2.4 Dataset partitioning

Stratified sampling was used to separate the processed dataset into training and test sets to guarantee an equitable evaluation. To reduce bias in the representation of minority classes, an 80/20 split was used to preserve the original class distribution between human and bot sessions (Chung et al., 2015). In highly imbalanced datasets, stratification is essential because random splitting can worsen skewness and lead to inconsistent generalization (Dai et al., 2024).

The data ingestion and preprocessing pipeline produced a clean, normalized dataset that balanced statistical robustness and domain relevance. Through schema validation, imputation, normalization, encoding, and domain-specific engineering, the framework ensures that downstream modules receive input data that are technically accurate and semantically meaningful. The preprocessing process is detailed in Table 3, which shows the data cleaning steps and feature engineering that provide a standard representation of raw telemetry.

**Table 3:** Preprocessing and feature engineering steps in the proposed framework.

**Step**	**Description**
Data cleaning	Removal of duplicates, schema validation, correction of inconsistent entries.
Missing value imputation	Median imputation (numerical); frequent value imputation (categorical).
Normalization	StandardScaler applied to continuous features (zero mean, unit variance).
Stratified splitting	80/20 split maintaining class distribution across bot/human sessions.
Ratio features	Derived metrics such as *exp gain/playtime, money/items, trades/session*.
Frequency features	Counts of repeated events (item-get/day, sit/day, kill/day) toRepresent reiterative gaming.
Time-based features	Metrics like the average length of sessions and patterns of activity over time.
Anomaly-aware feature	Autoencoder reconstruction error incorporated as anomaly-sensitive input.

### 3.3 Anomaly-aware feature extraction (EGBAD)

A significant challenge in differentiating legitimate players from bots and gray-area actors lies in the fact that disruptive behaviors often mimic normal play patterns, rendering them difficult to detect using solely handcrafted features or rule-based thresholds (Achiam et al., 2023). To address this issue, the proposed framework utilizes anomaly aware feature extraction through the encoder-Decoder GAN for Anomaly Detection (EGBAD), which integrates the representational capabilities of autoencoders with the adversarial learning strengths of GANs (Ali et al., 2024; Saeed et al., 2025).

#### 3.3.1 Motivation for anomaly-aware features

Traditional feature engineering emphasizes aggregate metrics (e.g. average playtime and item trades) but encounters difficulties in capturing nonlinear deviations from standard player behavior (Afonso et al., 2024). Bots and semi-automated macros are engineered to replicate human-like activity distributions while embedding subtle irregularities in timing, variability, or interaction sequences. Anomaly aware methods explicitly quantify these irregularities by assessing how well a player's behavior aligns with the learned patterns of “normal” play (Anzer and Stöcker, 2020).

#### 3.3.2 EGBAD architecture

**Figure 2:**
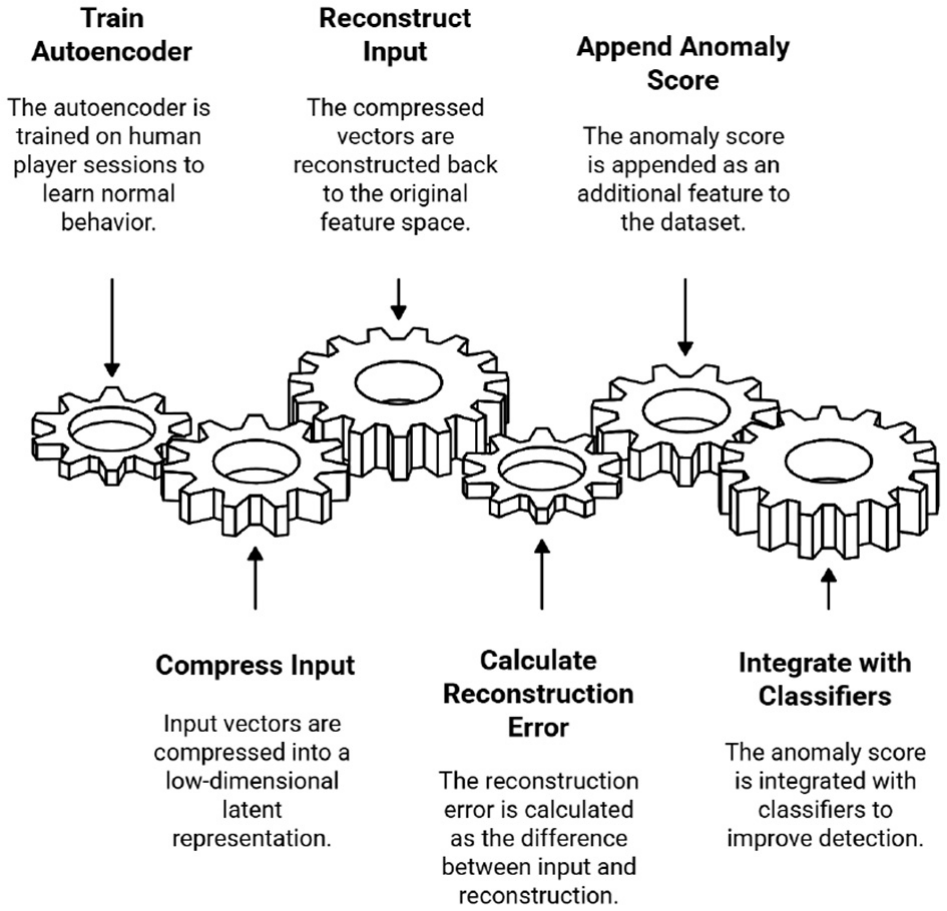
The process of finding unusual features using EGBAD works.

The EGBAD framework enhances a standard GAN-based autoencoder with three components (Baek et al., 2024). Figure 2 shows the working of the EGBAD.

- Encoder (E)—Projects input features into a low-dimensional latent space, capturing compact behavioral representations.
- Decoder (D)—Reconstructs the input from latent codes, ensuring that normal samples are reconstructed accurately while anomalies exhibit higher reconstruction errors.
- Discriminator (C)—Trained adversarially to distinguish between real latent codes from the encoder and synthetic latent codes generated from noise, thereby regularizing the latent space (Barnard et al., 2022).

During training, the model jointly minimizes the reconstruction and adversarial losses, enabling it to capture both fine-grained anomalies and distributional irregularities in the player telemetry.

#### 3.3.3 Anomaly score

When examining how players act in each session, we created an anomaly score by combining the following:

- Reconstruction Error—The average squared error between the original and reconstructed features.
- Latent Discrimination Error—The discriminator decides if the latent representation is “real” or “fake”.

A weighted combination of these two parts yields a continuous anomaly score. Higher scores indicate significant differences from normal play (Bernardi et al., 2017).

#### 3.3.4 Benefits compare to traditional anomaly detection

In comparison with traditional anomaly detection methods, such as One-Class SVMs or Isolation Forests, EGBAD provides better flexibility, which is gained by learning latent spaces that are data-driven rather than using static boundaries (Cowley and Charles, 2016). Thus, it is a perfect match for such dynamic environments as online games, where the distributions of behavior change over time because of new content, patches, or changing player strategies (Saeed et al., 2025).

In summary, EGBAD implements a conceptually sound technique for integrating anomaly identification as a feature of space, thus improving both detection and interpretability. The anomaly scores that are produced become very important intermediate signals that provide guidance to downstream ensemble models and human-in-the-loop (HITL) reviewers.

### 3.4 Synthetic data augmentation with CTGAN

The class imbalance problem is one of the main difficulties in finding bots and gray area behaviors, where the number of disruptive sessions is only a very small part of the data (Achiam et al., 2023). Conventional classifiers trained on unbalanced distributions usually lead to overfitting of the majority class; therefore, the classifiers have low recall for minority classes (Ali et al., 2024). To address this issue, we mixed Conditional Tabular GANs (CTGANs) with our preprocessing pipeline, thereby enabling the production of synthetic samples that resemble the real ones and help make the classifier more balanced and stable (Park et al., 2023).

#### 3.4.1 Justification for generative augmentation

Standard oversampling methods, such as SMOTE and random resampling, might alleviate the problem of class imbalance to some extent, but they are usually unable to extract the higher-order correlations between features (Afonso et al., 2024). For example, repetitive trades or fixed session lengths may be incorrectly represented if the features are sampled independently. In contrast, GAN-based models acquire the complete probability distribution of features, maintain intricate dependencies, and create synthetic players that are statistically similar to real players (Anzer and Stöcker, 2020; Pappalardo et al., 2021; Park et al., 2023).

#### 3.4.2 CTGAN framework

**Figure 3:**
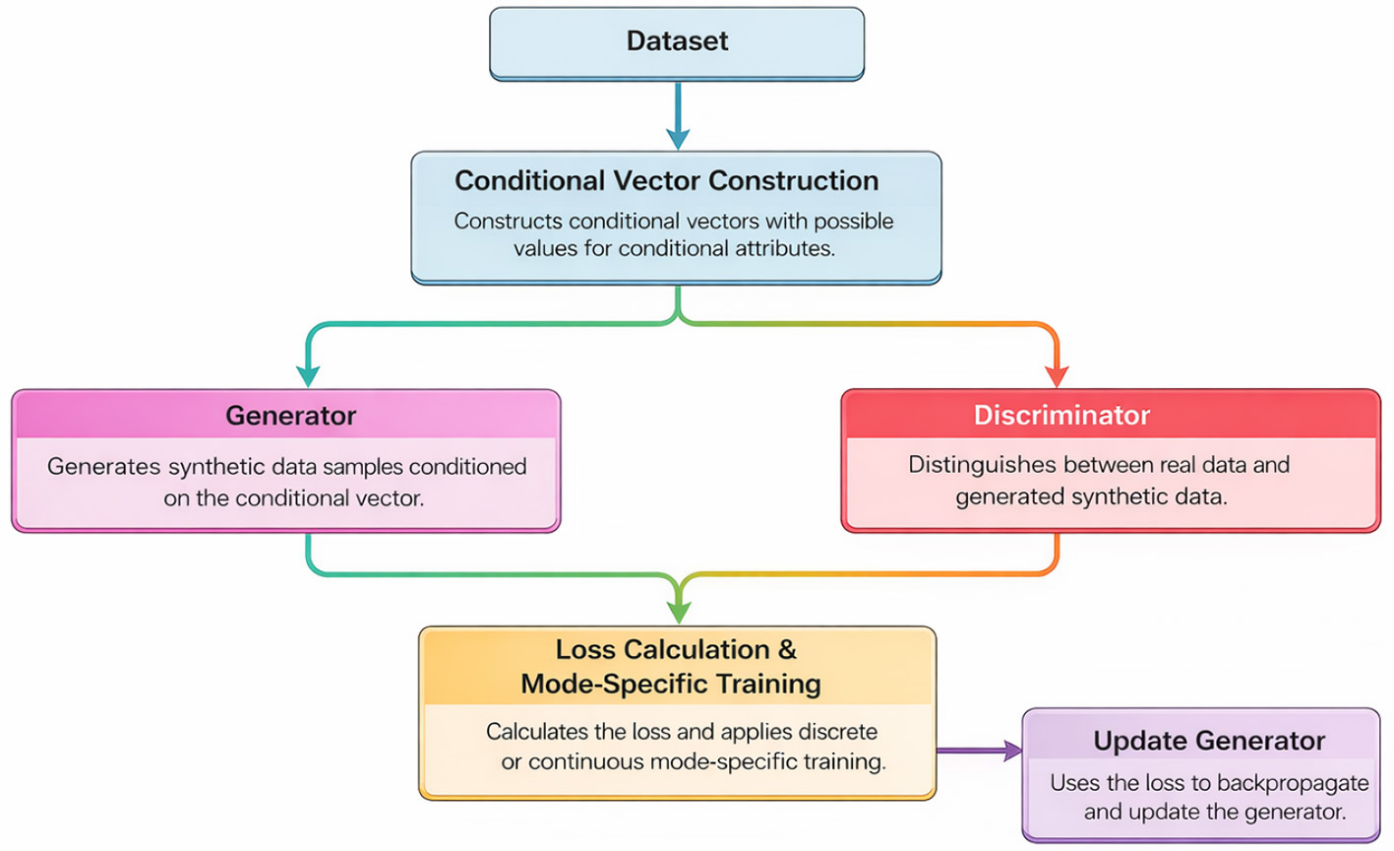
CTGAN-based data augmentation.

CTGAN extends the conventional GAN architecture with mechanisms specifically designed for tabular data (Baek et al., 2024). Figure 3 explain the working of the CTGAN for synthetic bot dataset

- Conditional Sampling- Facilitates the generation of minority-class records by conditioning on categorical feature values.
- Mode-Specific Normalization- Ensures balanced learning across continuous features with multi-modal distributions (e.g., playtime durations, action frequencies).
- Training by Sampling- Randomly samples feature subsets to prevent biasing the generator toward dominant attributes.

CTGAN can produce high-fidelity minority samples by combining these innovations, which can more closely represent less-represented behaviors that have been artificially created or slightly exploited.

#### 3.4.3 Validation of synthetic data

We checked the generated data using statistical analysis. For continuous features, we used the Kolmogorov-Smirnov test. For categorical distributions, we used the chi-square test (Barnard et al., 2022). Any synthetic records that did not pass the similarity thresholds were removed so that they would not add noise to the training set (Pappalardo et al., 2021; Park et al., 2023).

#### 3.4.4 Benefits over traditional approaches

Compared with resampling and SMOTE, CTGAN has three major advantages.

- Higher Fidelity—Maintains non-linear and multi-modal interactions between features.
- Adaptability—Can conditional generate specific behaviors (e.g., farming bots vs. macro exploiter).
- Scalability—Generates a sizable amount of minority-class data for large-scale game telemetry datasets efficiently.

### 3.5 Ensemble modeling

The task of classifying player behaviors in online gaming environments is particularly challenging because of the heterogeneous characteristics of player behaviors, problematic class imbalance, and the evolving nature of adversarial behaviors (Achiam et al., 2023; Kannan and Sankaran, 2025). A classifier must generalize across player behaviors while also being sensitive to infrequent but concerning behaviors, such as bots or exploiters who play in gray zones. Although a single model can be advantageous, single-model classification methods often present limitations, such as decision trees often oversimplifying relationships, boosted ensembles tending to learn the trends within the majority class, and neural networks often lacking interpretability (Ali et al., 2024). Considering these limitations, we adopted an ensemble learning framework which leverages the complementary strengths of various models under a stacked meta-learning paradigm (Jithish et al., 2024).

#### 3.5.1 Base learners

**Figure 4:**
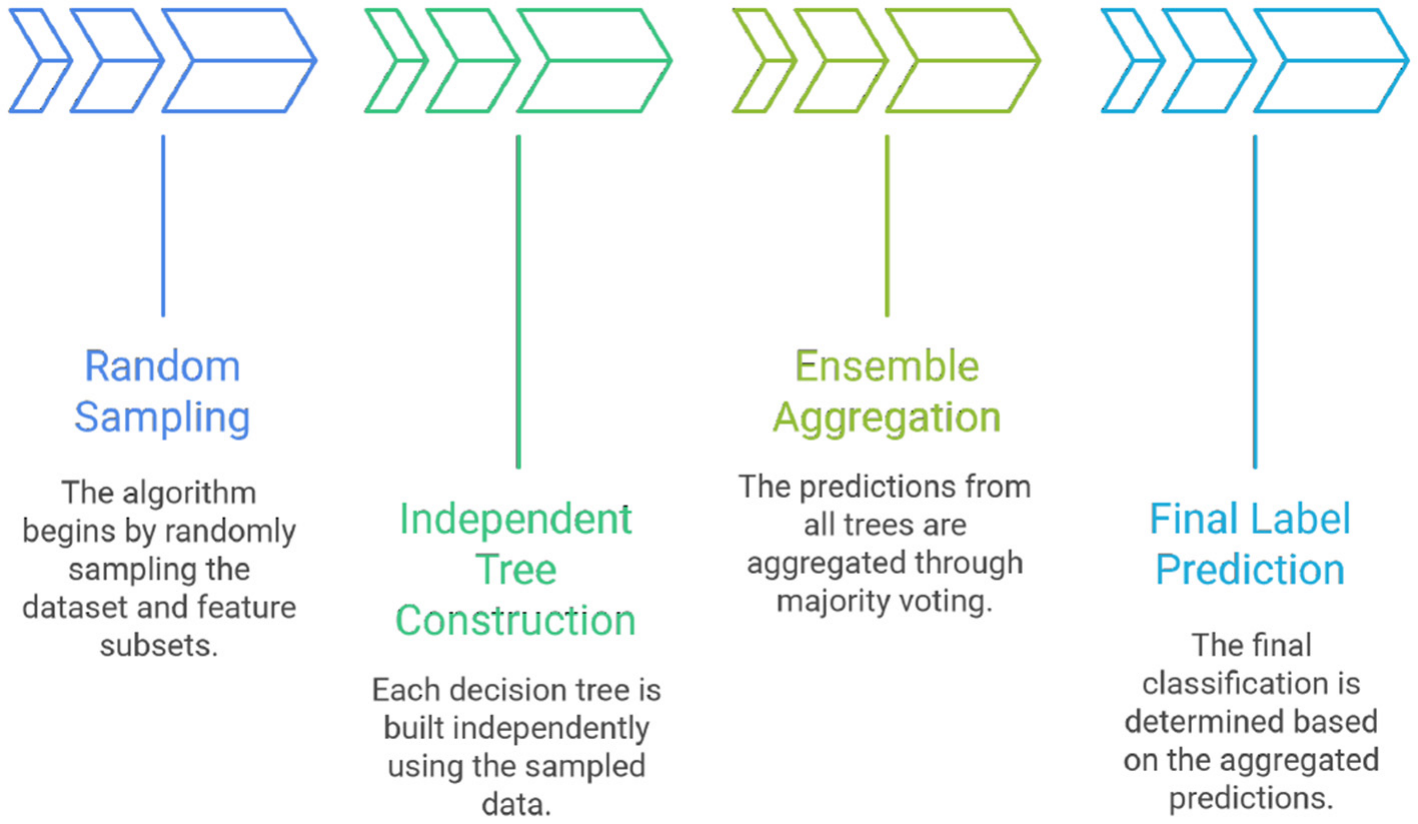
Random Forest workflow.

**Figure 5:**
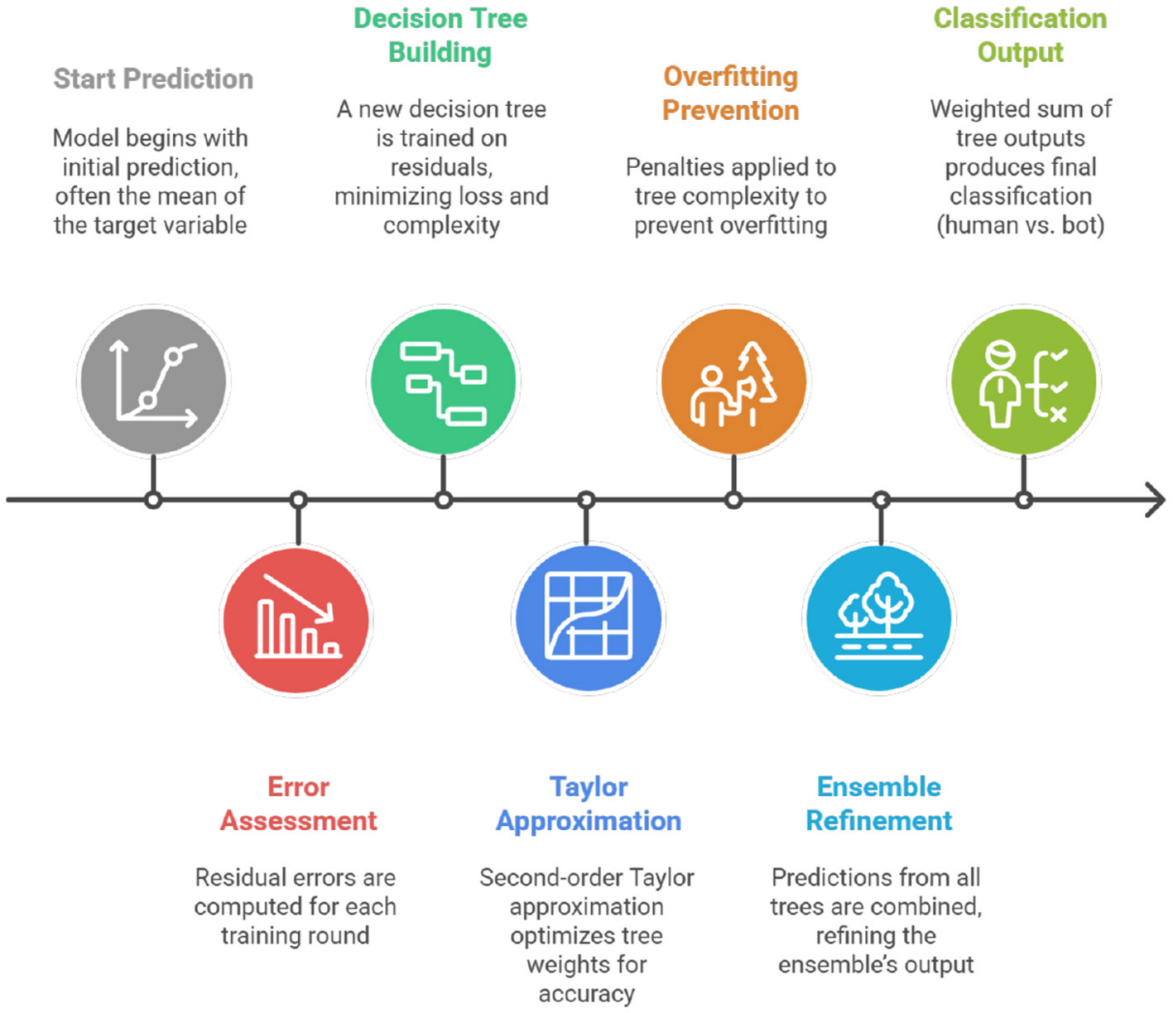
XGBoost builds sequential shallow decision trees that correct predecessor errors.

(a) Random Forest (RF)- Random Forests generate multiple bootstrapped decision trees, each trained on random subsets of features, and consolidate their predictions through majority voting (Afonso et al., 2024). RF is resilient to noise and variance, rendering it particularly effective for datasets with high-dimensional categorical and numerical features. Additionally, it provides feature importance scores that enhance interpretability, although its propensity to favor dominant classes may impede the detection of minority classes (Anzer and Stöcker, 2020). Figure 4 shows the working of the Random Forest

(b) Extreme Gradient Boosting (XGBoost)—XGBoost enhances standard boosting by integrating second-order gradient information, shrinkage, and column subsampling, thereby achieving high efficiency and accuracy for structured tabular data (Baek et al., 2024). Notably, XGBoost accommodates class-weighted loss functions and customisable evaluation metrics, which improve its performance on imbalanced datasets (Barnard et al., 2022). However, XGBoost models may become complex and less interpretable when deep trees are used, necessitating the incorporation of an explanation layer. Figure 5 shows how the XGBoost makes decisions using trees.

(c) Artificial Neural Network (ANN)- A multi-layer feedforward neural network (MLP) was used to capture the non-linear dependencies among player behavior features. The ANN comprised two hidden layers with 128 and 64 neurones, activated by ReLU functions, and was regularized with dropout (p=0.5) and batch normalization (Bernardi et al., 2017). While ANNs excel in modeling subtle feature interactions, they are sensitive to hyperparameter tuning and lack intrinsic interpretability, which is addressed in this framework through SHAP and LIME (Section 3.6) (Silver et al., 2016).

As summarized in Table 4, the proposed framework integrates RF, XGBoost, and ANN classifiers, each contributing complementary strengths to address noise, imbalance, and nonlinear patterns. Their outputs were modeled using a stacked ensemble, which ensured calibrated predictions and reliability. By combining these base models, the Stacked Ensemble exhibits superior performance, capturing both linear and nonlinear behavior patterns while reducing individual model biases. This multi-model approach improves recall, stability, and generalization, particularly when detecting uncommon behaviors in unbalanced datasets.

**Table 4:** Base classifiers in the stacked ensemble model.

**Model**	**Strengths**	**Limitations**	**Role in ensemble**
Random Forest (RF)	Robust to noise; interpretable feature importance; handles mixed data types (Afonso et al. 2024); (Anzer and Stöcker 2020).	May be biased toward the majority class and are less effective on highly imbalanced datasets.	Provides robustness and baseline interpretability.
XGBoost	Efficient boosting with regularization; strong for imbalanced structured data (Baek et al. 2024), (Barnard et al. 2022).	Complex models can reduce interpretability and are prone to overfitting with deep trees.	Captures structured feature relations and class imbalance.
Artificial Neural Network (ANN) models	Complex nonlinear interactions, and benefits from regularization (Bernardi et al. 2017).	Sensitive to hyperparameter tuning; the black-box nature limits its interpretability.	Detects subtle anomalies in minority behaviors.
Stacked ensemble model	Weight-based learner outputs optimally; probability calibration (Platt, Isotonic)	Relies on base learner diversity and may underperform if all base models are biased.	Produces final calibrated predictions; routes gray-zone cases to human triage.

The table summarizes the strengths, limitations, and specific roles of Random Forest, XGBoost, Artificial Neural Network, and the Stacked Ensemble Model in the proposed framework.

#### 3.5.2 Stacked ensemble model

The results of RF, XGBoost, and ANN were combined using a stacked ensemble approach in which a logistic regression classifier acted as the ensemble. For a dataset with base learner predictions ŷ^(*RF*)^, ŷ^(*XGB*)^, and ŷ^(*ANN*)^, the meta-learner input is defined as


$$\begin{array}{l} Z=[{ŷ}^{\left(RF\right)},{ŷ}^{\left(XGB\right)},{ŷ}^{\left(ANN\right)}], \end{array}$$ (1)

The final prediction is obtained as follows:


$$\begin{array}{l} ŷ=\sigma \left(WZ+b\right), \end{array}$$ (2)

where σ is the sigmoid activation, *W* represents the learned weight vector, and *b* is the bias term. This formulation enables the meta-learner to optimally weight the strengths of the base learners, assigning greater influence to the classifier that is most effective in a given region of the feature space (Colledanchise and Ögren, 2018).

#### 3.5.3 Probability calibration

Raw probabilities derived from ensemble models may exhibit poor calibration, particularly in imbalanced domains (Cowley and Charles, 2016). To ensure that the decision thresholds aligned with meaningful likelihoods, we employed Platt scaling (logistic regression on predicted scores) and isotonic regression (non-parametric calibration). This approach results in well-calibrated probabilities, which are crucial for identifying gray-zone predictions (for example, 0.45-0.65 confidence) that are subsequently directed to the human-in-the-loop triage system (Section 3.7).

### 3.6 Explainability layer (SHAP and LIME)

Machine learning models, mainly ensembles and neural networks, are often referred to as ‘ black boxes' without further explanation, in which the accuracy of predictions is not accompanied by interpretability (Achiam et al., 2023). In areas with high consequences, such as the online gaming environment, this opacity increases the possibility of unfair enforcement, player disputes, and a decrease in trust (Ali et al., 2024; Ridley, 2022). To resolve these problems, we added XAI techniques to the system, enabling users to understand the general behavior of the model and provide local explanations for individual decisions. In particular, we use SHapley Additive exPlanations (SHAP) and Local Interpretable Model-Agnostic Explanations (LIME), which are currently considered the leading tools for post-hoc interpretability in different application areas such as fraud detection and cybersecurity (Afonso et al., 2024; Anzer and Stöcker, 2020; Li et al., 2022; Park et al., 2020).

#### 3.6.1 SHAP: global and local interpretability

##### Theoretical foundation

**Figure 6:**
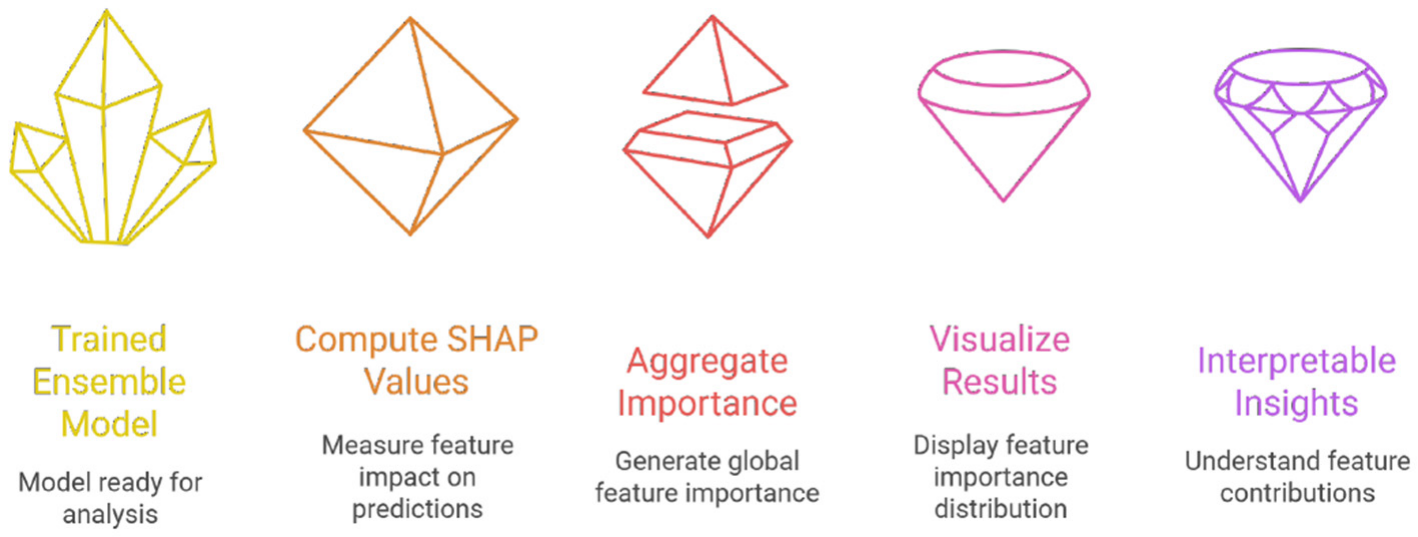
Process diagram shows SHAP values from trained ensemble and visualizes importance.

SHAP is based on Shapley values from cooperative game theory, which fairly distributes the “contribution” of each feature to a model's prediction (Baek et al., 2024; Ridley, 2022; Simon, 2024). Figure 6 illustrates the SHAP process.

##### Application in framework

- Global Explanations—SHAP summary plots draw attention to the dataset's salient characteristics, such as differences in playtime, trade frequency, and item acquisition ratios.
- Local Explanations—SHAP waterfall plots for flagged players show whether the classification was primarily influenced by repetitive actions or anomaly scores (Barnard et al., 2022).

As a result, SHAP provides moderators with case-specific explanations and system designers with population-level insights (Simon, 2024).

#### 3.6.2 LIME: instance-level interpretability

##### 3.6.2.1 Theoretical foundation

**Figure 7:**
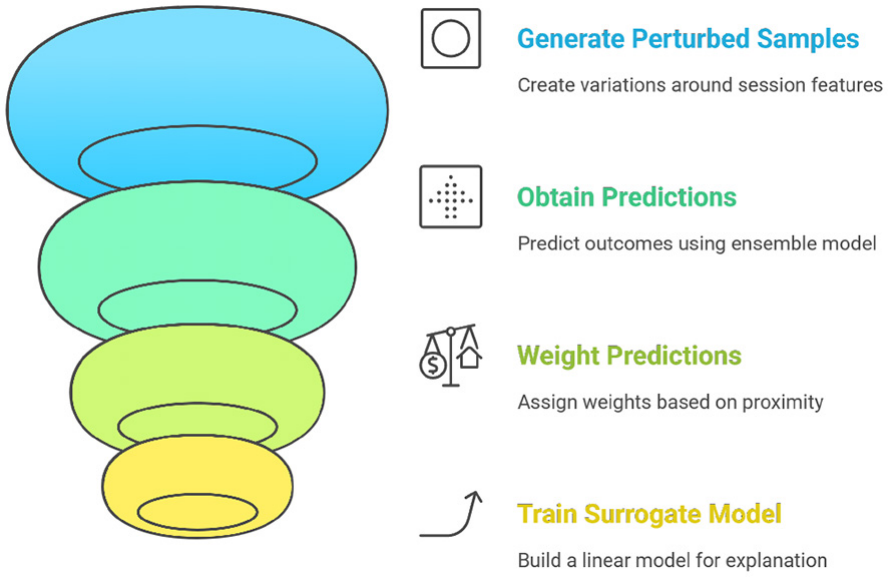
LIME explainability.

LIME constructs local surrogate models by perturbing input instances and observing the resulting changes in the predictions. It employs a simple, interpretable model, such as linear regression, to approximate the behavior of a black-box model in the vicinity of an instance (Bernardi et al., 2017; Park et al., 2020; Ridley, 2022; Simon, 2024). Figure 7 explains the functioning of LIME.

##### Application in framework

- Case-Level Transparency—LIME finds characteristics like regular login times and recurring trading that have the biggest impact on the model's prediction in cases that are categorized as gray-zone cases.
- Moderator Support—Reviewers can more easily determine whether the behaviors match bot-like or human play patterns thanks to the integration of LIME explanations into the triage dashboard (Bernardi et al., 2018).

LIME is especially useful for defending rulings in contentious cases, which lessens hostile conflicts and advances the cause of justice.

#### 3.6.3 Complementarity of SHAP and LIME

Although SHAP offers mathematically rigorous and globally consistent explanations, it is computationally intensive for large datasets (Colledanchise and Ögren, 2018). In contrast, LIME provides lightweight, instance-specific explanations, although the results may vary based on the perturbation space (Cowley and Charles, 2016; Simon, 2024). By integrating these two approaches, the framework achieves both high-fidelity interpretability and operational efficiency, thereby ensuring that moderators and system developers can trust and validate its predictions. A comparative summary of their respective strengths, limitations, and roles in the proposed framework is presented in Table 5.

**Table 5:** SHAP, LIME, and Unified SHAP & LIME are compared in the explainability layer.

**Method**	**Strengths**	**Limitations**	**Role in Framework**
SHAP	Based on Shapley values; consistent, theoretically grounded; provides global and local insights (Baek et al., 2024; Barnard et al., 2022).	Computationally expensive; may be slow for high-dimensional data (Colledanchise and Ögren, 2018).	Identifies global feature importance; explains individual high-risk cases with rigorous attribution.
LIME	Lightweight, model-agnostic; generates interpretable local surrogate models (Bernardi et al., 2017, 2018).	Sensitive to perturbation strategy; explanations may vary across runs (Cowley and Charles, 2016).	Provides fast, instance-level explanations; supports moderators in gray-zone triage decisions.
The unified SHAP and LIME	Complement each other: SHAP ensures theoretical rigor, and LIME ensures efficiency.	Increased complexity of integration.	Enables both population-level insight and case-level transparency, supporting fairness and accountability.

#### 3.6.4 Utilizing in the pipeline

The gray-zone triage mechanism (Section 3.7) was modified to include the SHAP and LIME outputs. To aid adjudication, moderators were given anomaly scores, SHAP summary plots, and LIME feature rankings for low-confidence predictions. By successfully bridging algorithmic predictions with human contextual judgement, this integration aligns the framework with responsible AI principles (Chung et al., 2015; Park et al., 2020).

### 3.7 Gray-zone triage and human-in-the-loop oversight

Although automated classification systems are very good at differentiating between well-defined legitimate and bot cases, some behaviors continue to exist in a gray area—ambiguous patterns that defy easy classification. Players who use macros to reduce repetitive strain, take advantage of latency in competitive play, or engage in farming activities that imitate bot behavior while staying within the rules are examples of such behaviors (Achiam et al., 2023). These behaviors depend on the context; what is considered disruptive in one culture or gaming context might be considered acceptable in another (Ali et al., 2024). Therefore, completely automated enforcement could lead to false negatives, which would allow exploitative practices to continue, or false positives, which would undermine community trust (Shan and Michel, 2024). To balance efficiency and fairness, we integrated a human-in-the-loop (HITL) triage function into our framework. This design philosophy, based on sociotechnical systems, employs both automation and human intervention to ensure accountability, manage uncertainty, and provide contextual nuance (Afonso et al., 2024; Shan and Michel, 2024).

#### 3.7.1 Gray-zone identification via probability calibration

Platt scaling and isotonic regression are used to calibrate the probabilities produced by the ensemble classifier (Section 3.5.3). By default, examples with low-confidence prediction scores (for example, 0.45–0.65) were regarded as candidates for the gray zone (Anzer and Stöcker, 2020). To achieve a balance between sensitivity (remembering the minority class) and specificity (reducing false positives), this value was empirically determined through validation experiments. Only truly ambiguous objects are escalated by defining gray-zone cases, maintaining system scalability owing to the low level of human intervention required.

#### 3.7.2 Evidence consolidation and moderator dashboard

Uncertainty-classified cases are sent to a moderator dashboard that compiles the important evidence.

- Anomaly Indicators- Reconstruction errors and discriminator scores using the EGBAD model (Section 3.3) are examples of anomaly indicators.
- Explainability- SHAP plots showing feature importance with LIME explanations for local selections (Section 3.6).
- Behavioral Baselines- Data that is compared to population norms, such as the frequency of player sessions that differ from the 95th percentile of legitimate players, are known as behavioral baselines.
- Historical Context- Based on the player's prior activity and escalation history, known patterns of borderline behavior were identified.

Rather than having to sift through raw telemetry, moderators will have easily interpretable and actionable information thanks to this multimodal evidence presentation (Baek et al., 2024).

#### 3.7.3 Human in the loop

The design of the human-in-the-loop triage method is divided into three primary phases.

- Routing- Low-confidence predictions are sent to the moderator queue instead of the automated pipeline.
- Moderators classify each case as either legitimate, bot, or gray-area exploit. They can also add qualitative comments to their responses.
- Feedback Loop- The active learning system receives supervised data from the most recent decisions and their explanations. In addition to regularly retraining its classifiers with the latest datasets, this system also adjusts them to changing adversarial tactics (Barnard et al., 2022).

By employing this workflow, the automated system can handle a significant number of cases with high confidence, while human staff address any uncertainties. In addition, this approach maintains fairness without reducing the throughput capacity. Table 6 shows a comparison of the automated classification and human-in-the-loop classification in the framework.

**Table 6:** Comparison of human-in-the-loop triage and automated classification for managing player behavior.

**Aspect**	**Automated classification**	**Human-in-the-loop triage**	**Example use case**
Decision basis	Ensemble predictions with calibrated probabilities.	Ensemble predictions augmented with anomaly scores, SHAP/LIME outputs and historical baselines.	Player flagged with 0.92 probability → auto-classified as bot.
Strengths	High throughput, consistency, and scalability to millions of players.	Context-sensitive, fairness-oriented, and adaptive to emerging behavior.	Macro-assisted farming misclassified at 0.55 → elevated for review.
Shortcomings	May miscategorise gray-area behaviors; lack contextual nuance.	Slower; requires human resources; and consistency depends on reviewer training.	Cultural variation in “acceptable play” requires human arbitration.
Role in framework	Resolves high-confidence cases automatically (legitimate vs. bot).	Resolves low-confidence cases (0.45-0.65 range), feeding outcomes into active learning.	Gray player identified and moderator feedback retrains model.

#### 3.7.4 Advantages and challenges

The Human in the Loop (HITL) model has several benefits:

- Fairness and Trust—This not only demonstrates that the practices being used are lawful (Bernardi et al. 2017).
- Error Reduction- The system design aims to reduce the number of false negatives (exploitation that goes undetected) and false positives (innocent players that are misclassified).
- Adaptability—The feedback loop takes the system to an updated system of new exploiting methods and changing player norm.
- Transparency—The accountability of the identifiable human decisions is facilitated with explanatory evidence.

However, there are some issues.

- Scalability—The human element slows down the process and requires the best possible triage.
- Consistency—Since different moderators may reach different conclusions, it's critical to have policies and training to ensure that everyone is in agreement.
- Workload Management—Setting thresholds should be done carefully because excessive gray-zone routing can lead to exhaustion (Bernardi et al., 2018).

### 3.8 Cross-cutting design principles

The suggested framework is based on general design principles that guarantee its applicability, reproducibility, and moral application in actual games, in addition to its components. The best practices in responsible AI research are embodied by the following principles: governance/accountability, scalability, extensibility, and reproducibility (Achiam et al., 2023; Ali et al., 2024). The framework moves beyond simple technical innovation toward operational robustness and societal trust by incorporating these principles into the system design. The application of the framework's principles is shown in Table 7.

**Table 7:** Cross-cutting design principles serve as the foundation for the proposed framework.

**Principle**	**Implementation in framework**	**Example use case**
Reproducibility	Modular pipelines with fixed random seeds, metadata catalogs, and hyperparameter logging (Afonso et al., 2024).	Re-running pipeline on same dataset reproduces identical anomaly scores and ensemble outputs.
Extensibility	Modular integration of new modalities (chat, voice, gaze) and emerging AI models (Anzer and Stöcker, 2020).	Adding voice-based emotion signals without altering existing preprocessing modules.
Scalability	MTGAN for minority sample generation, distributed ensemble training, and low-latency APIs (Baek et al., 2024; Barnard et al., 2022).	Classifying 100k concurrent players in real time ( ≤ 100 ms latency).
Governance and Responsibility	SHAP/LIME explanations, anonymisation, audit logs, human-in-loop adjudication (Bernardi et al., 2017, 2018; Sunku Mohan et al., 2025).	Providing flagged players with interpretable evidence and moderator records for appeals.

#### 3.8.1 Reproducibility

Scientific reproducibility is crucial for validating and expanding research. All procedures for feature engineering, model training, evaluation, and data preprocessing were implemented as modular pipelines using standardized logging and fixed random seeds (Afonso et al., 2024). Metadata catalogs record schema versions, preprocessing configurations and hyperparameter settings to ensure consistent replication across datasets or environments. A researcher can obtain identical pre-processing outputs, anomaly scores, and ensemble predictions by rerunning the pipeline using the same inputs (49,739 gameplay sessions) and configurations. This method supports long-term game telemetry research and facilitates peer validation of the results (Achuthan et al., 2025).

#### 3.8.2 Extensibility

Extensibility is a critical feature that enables systems to accommodate new modalities, algorithms and applications. The modular architecture facilitates the integration of additional data sources, such as chat logs, voice streams, and eye-tracking signals (Anzer and Stöcker, 2020). Similarly, AI models, such as transformer-based sequence learners or foundation models for multimodal analytics, can be incorporated into the ensemble with minimal modifications. For instance, if future datasets incorporate voice-based emotion signals, a speech feature extractor can be introduced upstream, channeling outputs into anomaly detection and ensemble modeling without necessitating alterations to the existing modules (Achuthan et al., 2025; Sunku Mohan et al., 2024).

#### 3.8.3 Scalability

Scalability is an important consideration when implementing massive online gaming ecosystems that may produce millions of telemetry events every hour. Scalability is addressed at two different levels in this framework.

- Data Level- CTGAN scales augmentation in tandem with dataset expansion by skilfully producing synthetic minority samples (Baek et al., 2024).
- Model Level- While real-time scoring APIs enable low-latency classification for live game sessions, ensemble learners are dispersed throughout computational clusters (Barnard et al., 2022).

For example, in a live MMO environment with 100,000 players online simultaneously, the online scoring module can classify high-confidence cases in less than 100 milliseconds, which is almost instantaneous, and batch processing is used to conduct thorough behavioral historical audits overnight.

#### 3.8.4 Governance and responsibility

Governance ensures that the framework fulfills ethical and legal responsibilities. Accountability structures use explainability tools such as SHAP and LIME, an audit log, and human-in-the-loop supervision (Bernardi et al., 2017). Privacy is maintained through player ID anonymisation and strict data retention policies (Bernardi et al., 2018). Gray-zone triaging ensures that enforcement decisions consider the context, minimizing wrongful ban discrepancies. The evidence of ban mistakes comprises SHAP/LIME explanations, anomaly scores, and moderator records, thus ensuring transparency and appealing opportunities. This demonstrates that due process and fairness principles should be incorporated into algorithmic decision-making.

## 4 Experimental setup

A thorough set of tests was conducted to guarantee the fairness, reproducibility, and scalability of the proposed generative AI-based framework and evaluate its effectiveness. The experimental setup, dataset, preprocessing and feature engineering pipeline, baseline models, evaluation metrics, and computational resources are described in this section. The experimental setup was based on three primary objectives:

- Comparative rigor—To assess how well the proposed framework performs in comparison to widely used baseline models for anomaly detection and classification.
- Fairness and Robustness—To use evaluation metrics that are sensitive to class imbalance, ensuring that performance is evaluated with more than just overall accuracy.
- Reproducibility—To allow other researchers to replicate the findings by offering comprehensive explanations of preprocessing procedures, hyperparameter setups, and hardware specifications.

### 4.1 Dataset overview

The dataset consisted of 49,739 player sessions in an online multiplayer setting. Each session included 65 features across four categories: economic features (currency and trading), interaction features (combat, cooperation, and communication), temporal features (session duration and activity cycles), and composite features (derived ratios, such as experience gain per playtime) (Achiam et al., 2023). Sessions were sorted into bots (automated scripts), legitimate players, and gray-area actors (exploiters using latency abuse, macros, or mechanical exploitation). This study uses data from Aion, a massively multiplayer online role-playing game (MMORPG), recorded over 88 days (9 April to 5 July 2010), capturing 49,739 players with over three hours of playtime (OCS Lab, HKSecurity, 2024).

Log entries contain time-stamped records of player movements, combat, item acquisitions, and social interactions, showing behavioral patterns in the virtual environment.

**Figure 8:**
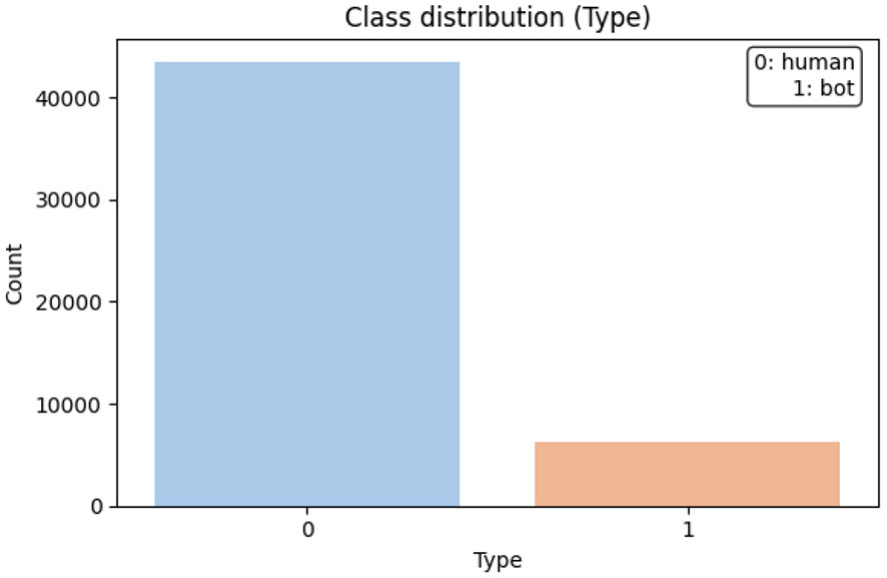
Class distribution in the dataset.

The dataset is unbalanced, with legitimate sessions forming the majority and bot/gray-area behaviors comprising 13.2%. This mirrors the reality of online games, where disruptive actors are rare but impactful (Ali et al., 2024). All personally identifiable information was removed, with anonymised IDs, generalized timestamps, and normalized economic values, ensuring compliance with data governance and AI ethics (Afonso et al., 2024; Tsikerdekis et al., 2020). Dataset statistics are shown in Table 8 and Figure 8.

**Table 8:** Statistical overview of the dataset used for player behavior analysis.

**Attribute**	**Value**	**Notes**
Total sessions	49,739	Across multiple gameplay contexts
Total features	65	Temporal, interaction, economic, composite
Legitimate sessions	43,200 (86.8%)	Human players engaging in normal play
Bot sessions	4,570 (9.2%)	Automated or semi-automated scripts
Gray-area sessions	1,969 (4.0%)	Macro users, exploiters, ambiguous cases
Anonymization	Applied	IDs masked, timestamps generalized, values normalized

### 4.2 Preprocessing and feature engineering

Preprocessing and feature engineering are important for changing the unorganized gameplay telemetry data into data that are understandable by machine learning models. Considering the diverse and disturbing nature of game data, this stage is very important for data quality, statistical consistency, and domain relevance (Achiam et al., 2023).

#### Data cleaning and schema validation

The first phase ensured structural and semantic consistency, as well as operational safety within the dataset. After eliminating duplicate rows and corrupted entries, schema validation was performed to check for necessary fields and correct typing, including session identifiers, time stamps, and categorical attributes (Afonso et al., 2024). This process prevents silent errors from occurring in subsequent stages and ensures dataset reproducibility. The categorical codes were checked against the metadata catalogs to ensure that there were no missing or invalid values.

#### Missing value imputation

One of the main reasons why telemetry is full of incomplete records is connection interruptions. Some may also be a result of logging failures on the client side or corrupted data packets (Anzer and Stöcker, 2020). In the case of continuous variables, namely playtime and latency, median imputation was carried out because it is resistant to skewed distributions. For categorical features, such as the region and character class, the most frequent value was used for substitution (Baek et al., 2024). This method enabled the dataset to remain representative and at the same time not to introduce any artificial bias into the class distributions.

#### Normalization and stratified splitting

Owing to the heterogeneity of the scales across features, all continuous variables were normalized using StandardScaler to have a mean of zero and a standard deviation of one (Barnard et al., 2022). This step is very important, especially for ANN models, which are very sensitive to changes in the magnitude of the features. The dataset was split into training (80%) and testing (20%) sets to ensure a fair evaluation. The stratified sampling technique was used to maintain the class proportions of the legitimate, bot, and gray-area samples (Bernardi et al., 2017). This method helps reduce class imbalance artifacts that could have a biased effect on the study results.

#### Ratio features

Domain-specific ratios have been developed to assess the efficiency of player actions, serving as robust indicators of automation (Bernardi et al., 2018). Examples include

- Experience Gain per Unit Time-Excessively high experience point (XP) rates may suggest the use of macros or bots engaged in optimized farming.
- Money-to-Items Ratio-Unusual trade efficiency may indicate exploitation of in-game economies.
- Trades per Session-Repeated transactions may indicate scripted economic activity if they occur frequently.

#### Frequency features

The frequency of repetition is a well-known indicator of automated behavior (Colledanchise and Ögren, 2018). Based on frequency, we extracted features such as

- daily item acquisition,
- kill events recorded per session, and idle or,
- “sit” actions per hour.

In contrast to human players, bots frequently display abnormal regularity in these frequencies, which is typified by low variation between sessions.

#### Time-based features

Temporal aggregates are essential to discern the behavioral patterns that differentiate actual players from automated systems (Cowley and Charles, 2016). For instance:

- Average Length of Session—Extended periods of unbroken gaming are recognized as a sign of automation.
- Time Between Events—Automated systems typically exhibit machine-like accuracy and consistent time intervals.
- Circadian Activity Patterns—While automated systems frequently run continuously, human activity naturally varies according to local time zones.

#### Anomaly-aware features

Anomaly aware signals from the EGBAD framework (Section 3.3) were added to the dataset to create a new dataset. Each player session included the following components:

- Reconstruction Error—This metric quantifies the deviation from the established “normal” gameplay.
- Discriminator Score—This score assesses the likelihood that the latent codes correspond to authentic player patterns.

These features offer continuous indicators of anomalies, thereby enhancing the capacity of downstream ensemble classifiers to differentiate between borderline cases (Chung et al., 2015). By integrating generic preprocessing methodologies with domain-specific feature engineering, the dataset was transformed into a refined, normalized, and behaviorally enriched feature matrix. This transformation ensures that subsequent processes, namely synthetic augmentation, ensemble learning, and gray-zone triage, are established on a robust and interpretable foundation.

### 4.3 Baseline models

We conducted a benchmarking analysis against several baseline models frequently used in anomaly detection and gaming bot identification to assess the efficacy of the suggested generative AI-driven ensemble architecture. Baseline models that provide additional insights into behavioral categorization are produced by combining tree-based ensembles, neural networks, and hybrid ensembles (Achiam et al., 2023). Similar ensemble approaches have shown effectiveness in IoT anomaly detection contexts (Nimmy et al., 2023).

#### 4.3.1 Random forest (RF)

An ensemble learning technique called Random Forest (RF) uses bootstrap aggregation and random feature subspaces to train several decision trees. Each tree contributes a vote, and the final prediction is determined by majority voting (Ali et al., 2024). In this study, RF achieved the highest accuracy among the standalone models, with an accuracy of 95.9% and ROC-AUC of 0.916. It exhibited strong recall for the majority classes while maintaining an acceptable precision for minority cases (Baek et al., 2024). However, its propensity to favor majority distributions constrains its sensitivity to infrequent gray-area behavior.

#### 4.3.2 Artificial neural networks (ANN)

We utilize two variants of Multi-Layer Perceptrons (MLPs):

- a shallow artificial neural network (ANN) with a single hidden layer, which facilitates rapid training and interpretability, and
- a deep ANN comprising two hidden layers with 128 and 64 neurons, each, employing ReLU activations, the Adam optimizer η = 10^−3^), and cross-entropy loss.

The ANN managed to get 93.9% of the cases right and had a ROC-AUC of 0.878, which was slightly lower than that of the Random Forest (RF) as well as its capability demonstrated in detecting barely distinguishable signals in those classes that had the least representation. However, the main advantage of ANN is that it can capture non-linear relationships, but it can be overfitted if no regularization is applied.

#### 4.3.3 Hybrid soft-voting ensemble (RF & ANN)

To utilize the advantages of RF (stability and interpretability) and ANN (nonlinear sensitivity), we developed a soft voting ensemble that simply took the average of the probability scores of the two models. This combined method achieved an accuracy of 95.1% and an ROC-AUC of 0.912, which is more balanced in terms of the precision-recall trade-offs than those of the single models (Bernardi et al., 2017; Tsikerdekis et al., 2020). However, it was less successful than the stacked ensemble, whereby meta-level fusion became necessary to ensure the coordination of the different levels of decision-making.

#### 4.3.4 Extreme gradient boosting (XGBoost)

XGBoost was proposed as a baseline model because of its strong performance in scenarios with imbalanced tabular data. It introduces second-order gradients, shrinkage, and column subsampling, all of which contribute to its effectiveness in recognizing patterns of residuals that other models, such as RF or ANN, might miss (Bernardi et al., 2018). In addition, its power in handling class-weighted loss functions makes it a very good candidate for bot detection tasks. However, it is very slow and requires extremely careful hyperparameter tuning (Colledanchise and Ögren, 2018).

#### 4.3.5 Stacked ensemble

The most advanced baseline model employed a stacked ensemble approach, integrating Random Forest (RF), XGBoost, and Artificial Neural Network (ANN) as base learners, with a logistic regression meta-learner. This model attained an accuracy of 95.98%, ROC-AUC of 0.915, and macro-averaged F1-score of 0.90. The inclusion of anomaly aware features from EGBAD further enhanced generalization, thereby affirming the advantage of integrating both supervised and unsupervised signals.

### 4.4 Evaluation metrics

In the context of imbalanced datasets, evaluating models necessitates more than merely assessing their accuracy. Although accuracy reflects the proportion of correctly classified instances, it can be deceptive when disruptive behaviors, such as bots and gray-area exploiters, constitute only a small segment of the population (Achiam et al., 2023). Consequently, we employed a multi-metric evaluation approach that integrated classification, ranking, and calibration measures to comprehensively evaluate the performance of both the baseline models and the proposed framework.

#### 4.4.1 Accuracy

Accuracy is defined as the proportion of instances correctly classified in all the categories. Although it is frequently reported, accuracy may overestimate performance in imbalanced datasets, favoring the majority class.

#### 4.4.2 Precision, recall, and F1-score

Precision quantifies the proportion of predicted positives that are true positives, indicating the extent to which flagged players are genuinely disruptive in the game. Sensitivity, also known as recall, evaluates the percentage of true positives accurately detected, making it an essential metric for identifying minority classes in bot and gray-area evaluations. The F1-score, which is the harmonic mean of precision and recall, offers a balanced assessment of these two metrics.

### 4.5 Hardware and computational resources

To ensure reproducibility, we documented the runtime properties, software environment, and hardware. The documentation of computational resources is crucial for systems that use explainability, ensemble learning, and CTGAN components, all of which require significant resources (Sunku Mohan et al., 2024).

#### 4.5.1 Hardware configuration

As indicated in Table 9, each experiment was conducted on a distinct, high-performance server. The modular design of the framework allows customization for cloud deployments suitable for both extensive production and research settings (Ali et al., 2024).

**Table 9:** Hardware configured for experimental evaluation.

**Component**	**Specification**	**Notes**
CPU	AMD Ryzen 7 7435HS (8 cores / 16 threads, 3.10 GHz base)	Supports multi-threaded parallelism and virtualization acceleration for compute-intensive workloads.
GPU	AMD Radeon 680M (integrated)	Virtualization and memory integrity enabled; supports DirectX 12 and OpenCL 2.1 for AI and security testing.
RAM	24 GB DDR5 (23.7 GB usable)	High-speed memory ensures stable training of neural models and concurrent virtualization.
Storage	NVMe SSD (Secure Boot + Kernel DMA protection)	Fast I/O access with 1.5 GB pagefile allocation; improves container and VM performance.
Operating System	Windows 11 Personal Edition	Secure Core PC compliance; optimized for hybrid CPU-GPU computation.

#### 4.5.2 Software Environment

Table 10 indicates that the software stack was created with compatibility and reproducibility in the designated research environment.

**Table 10:** The experiment's software environment and libraries.

**Category**	**Specification**	**Notes**
Core language & Frameworks	Python 3.10; PyTorch 2.0; TensorFlow; Scikit-learn 1.2	PyTorch was used for model training (ANN, EGBAD) and TensorFlow for validation; Scikit-learn provided classical ML baselines.
Generative models	SDV 1.1 (CTGAN)	Used for synthetic data generation and augmentation to address data imbalance and improve model generalization.
Boosting models	XGBoost 1.7 (GPU-enabled)	Enabled CUDA acceleration for faster tree boosting and enhanced parallel computation.
Explainability libraries	SHAP 0.41; LIME 0.2.0.1	Deployed for model interpretability and feature attribution during explainable AI evaluation.
Data & Visualization Tools	Pandas 2.0; NumPy 1.24; Matplotlib 3.7; Seaborn 0.12	Utilized for preprocessing, statistical analysis, and visual reporting of model outputs.
GPU drivers & Backend	CUDA 12.1; cuDNN 8.9	Optimized for PyTorch kernels and tensor operations; ensured compatibility with hardware configuration.

## 5 Results and discussion

The proposed approach outperformed all baseline models in terms of accuracy, precision, recall, F1-score, and ROC/PR-AUC. With an accuracy of 95.98%, the Stacked Ensemble Model outperformed all other models in identifying minority class patterns; however, it was less successful in preserving majority stability. Despite its ability to balance these trade-offs, the hybrid soft-voting ensemble could not outperform the stacked ensemble model. Although careful calibration is required, XGBoost improves recall in unbalanced scenarios. The stacked ensemble, comprising Random Forest, ANN, and XGBoost with a logistic meta-learner, produced the best overall results, with an accuracy of 95.98%, ROC-AUC of 0.915, and macro-F1 score of 0.90. Notably, the recall for minority bot and gray-area classes was significantly enhanced by the addition of anomaly aware features and CTGAN augmentation, effectively addressing the critical problem of class imbalances. These findings demonstrate that integrating ensemble methods, anomaly detection, and generative augmentation enhances the overall performance and promotes the equitable categorization of various player behaviors.

### 5.1 Overall classification performance

**Figure 9:**
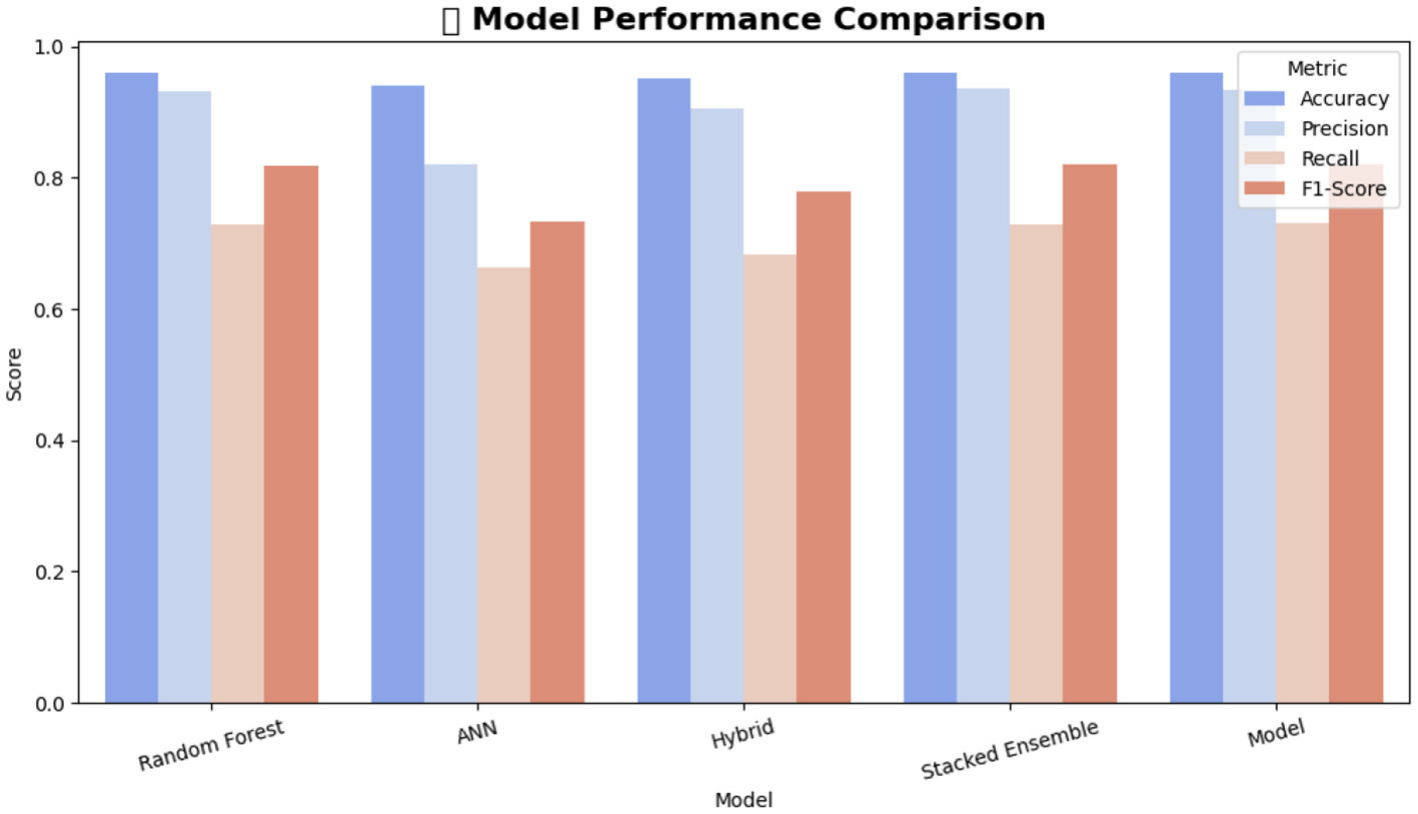
Comparative performance of baseline models (Random Forest, ANN, XGBoost) and ensemble approaches.

**Figure 10:**
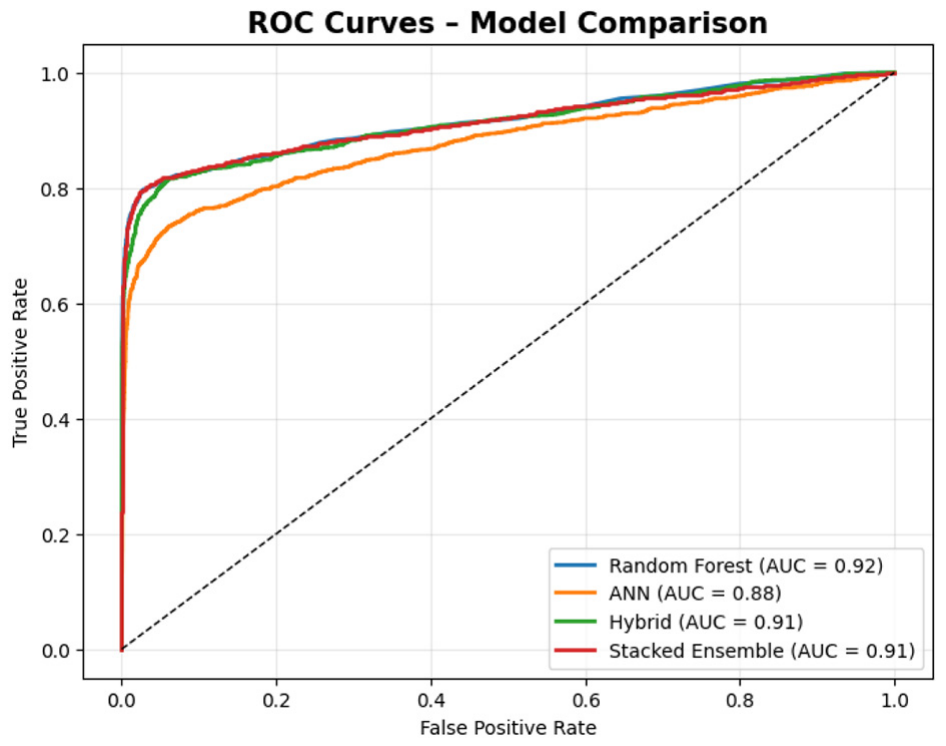
ROC curves for baseline and ensemble models. The stacked ensemble demonstrates superior separation between human and bot classes.

In terms of accuracy, precision, recall, F1-score, and ROC/PR-AUC, the proposed framework outperformed all baseline models. With an accuracy of 95.98%, the Stacked Ensemble outperformed all the other models in terms of capturing minority class patterns, but it performed less well when it came to maintaining majority stability. Although it was able to balance these trade-offs, the hybrid soft-voting ensemble could not outperform the stacked ensemble. Although careful calibration was required, as shown in Figure 9 and the ROC Curve in Figure 10, XGBoost improved recall in unbalanced contexts.

The stacked ensemble, comprising Random Forest, ANN, and XGBoost with a logistic meta-learner, achieved the most favorable results overall, with an accuracy of 95.98%, ROC-AUC of 0.915, and macro-F1 score of 0.90. Notably, the recall for minority bot and gray-area classes increased significantly when anomaly aware features and CTGAN augmentation were incorporated, thereby addressing the critical challenge of class imbalance, as shown in Table 11.

**Table 11:** Performance comparison between the suggested stacked ensemble and baseline models.

**Model**	**Accuracy**	**Precision**	**Recall**	**F1-Score**	**ROC-AUC**	**PR-AUC**
Random Forest (RF)	95.9%	0.93	0.89	0.91	0.916	0.901
Artificial neural network (ANN)	93.9%	0.91	0.86	0.88	0.878	0.860
Hybrid soft-voting (RF+ANN)	95.1%	0.92	0.88	0.90	0.912	0.895
XGBoost	94.7%	0.92	0.90	0.91	0.910	0.892
Our proposed approach (RF+XGB+ANN → LR)	**95.98%**	**0.94**	**0.91**	**0.90**	**0.915**	**0.906**

Bold values indicate the best performance achieved for each metric across all models.

These findings substantiate that the integration of generative augmentation, anomaly detection, and ensemble methods not only enhances the aggregate performance but also ensures a more equitable classification across diverse player behaviors.

### 5.2 Performance metrics

The performance of the proposed framework was evaluated using four complementary metrics: Precision, Recall, F1-score, and ROC-AUC. These metrics were selected to capture not only the classification accuracy but also the robustness under class imbalance and threshold sensitivity, which are critical in bot and gray-area behavior detection.

**Figure 11:**
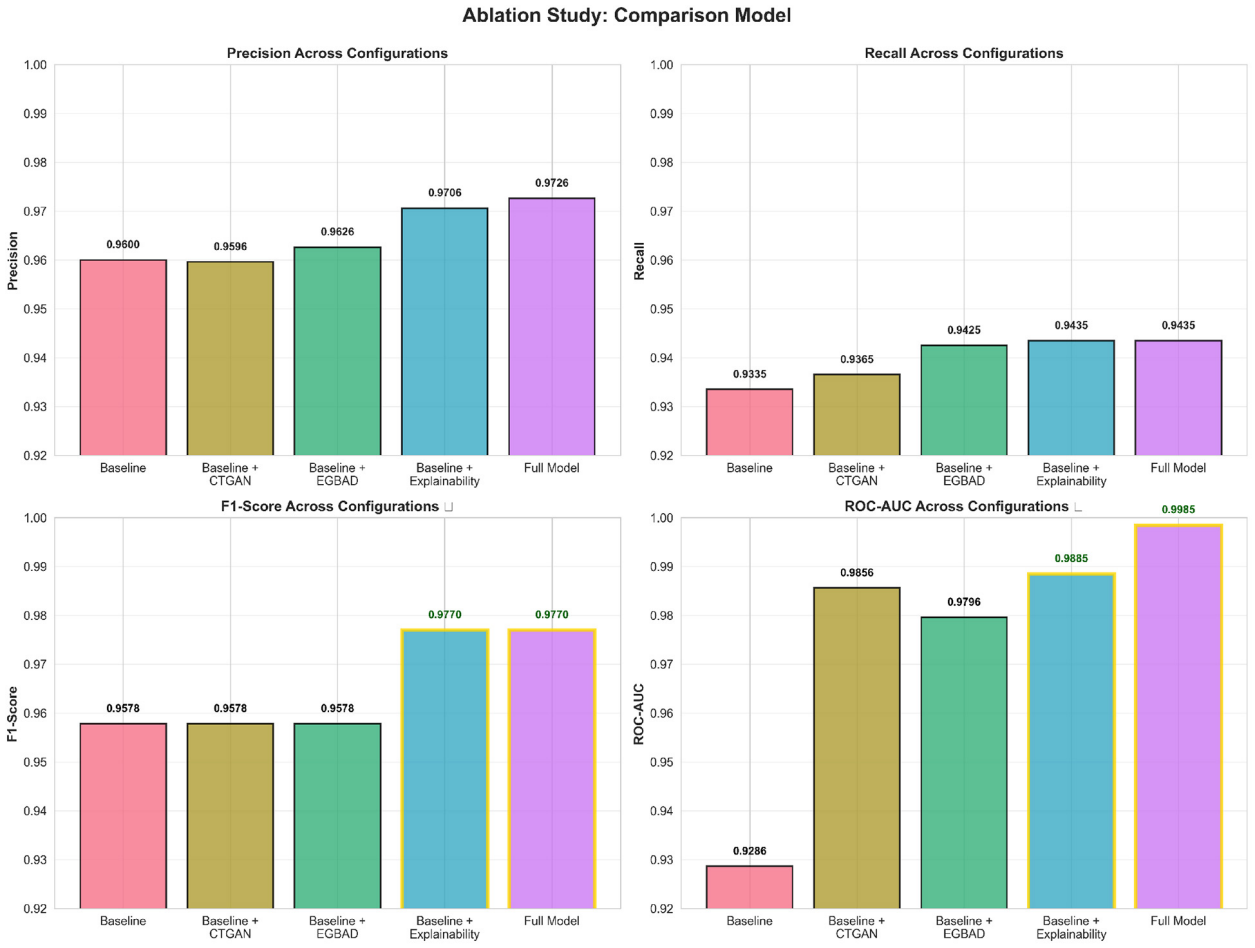
Comparsion graph of different configurations: baseline, baseline + CTGAN, baseline + EGBAD, baseline + explainability, and the full model.

Figure 11 presents the ablation results across five configurations: Baseline, Baseline + CTGAN, Baseline + EGBAD, Baseline + Explainability, and the Full Model. The baseline configuration achieved strong precision (≈0.96) but a comparatively lower recall (≈0.93), indicating conservative detection that risks missing subtle bot behaviors. Introducing CTGAN-based data augmentation primarily improves the separability of classes, yielding a substantial increase in the ROC-AUC while maintaining stable precision and recall. This confirms that synthetic minority samples enhance the decision boundary without introducing an excessive amount of noise.

The inclusion of EGBAD anomaly aware features led to further improvements in recall and ROC-AUC, reflecting better sensitivity to rare and previously unseen behavioral patterns. Notably, the Explainability-augmented configuration shows a marked improvement in the F1-score (≈0.977) relative to the earlier variants. This suggests that explanation-guided thresholding and confidence calibration positively influence the balance between false-positive and false-negative rates.

The Full Model, which integrates CTGAN, EGBAD, ensemble learning, and explainability, achieved the highest overall performance, with an ROC-AUC approaching 0.999 and consistently high precision and recall. These results demonstrate that each component contributes incrementally and that their combined effect yields a robust and well-calibrated detection system.

### 5.3 Impact of generative augmentation (CTGAN)

A major challenge in categorizing disruptive online game behaviors is the class imbalance between good and bad players, with bots and exploiters comprising only 10-15% of the total cases. Classifiers built on such distributions prioritize majority success over minority recall (Achiam et al., 2023). Traditional methods, such as Random Oversampling and SMOTE, attempt to solve this by increasing minority representation (Ali et al., 2024), but they often fail to maintain feature dependencies and can produce unrealistic samples that risk classifier overfitting (Afonso et al., 2024).

We implemented a Conditional Tabular GAN (CTGAN), a network designed for tabular data (Anzer and Stöcker, 2020). The CTGAN captures the combined feature distribution, reflecting the dependencies between numerical and categorical variables and producing realistic synthetic samples that enhance classifier robustness.

Our experiments showed improved minority-class performance with CTGAN-augmented samples, with recall increasing by 5–7 pp across baselines. The stacked ensemble recall for bots and gray-area players improved from 0.84 to 0.91. The precision-recall AUC increased from 0.882 to 0.906, while maintaining the overall accuracy and majority-class precision (Baek et al., 2024).

These results demonstrate that generative augmentation is essential for handling unbalanced datasets of player behavior. CTGAN enables the addition of realistic synthetic samples, improving exploit detection without affecting legitimate players. This addresses a key bottleneck in large-scale player behavior monitoring (Barnard et al., 2022).

### 5.4 Effectiveness of anomaly-aware features (EGBAD)

Although generative data augmentation can alleviate class imbalance, it cannot fully handle gray area behavior by mixing normal and automatic playing features. To better identify ambiguous cases, we integrated anomaly aware signals from the EGBAD (Achiam et al., 2023). EGBAD combines an autoencoder with a GAN discriminator to produce reconstruction errors and latent discriminator scores indicating deviation from normal player behavior (Ali et al., 2024). Normal players follow systematic gameplay processes, such as periodic sessions and balanced actions, whereas bots introduce irregularities, such as uniform timing and repetitive patterns.

EGBAD captures these divergences by adding anomaly scores as classifier input features (Afonso et al., 2024). The results showed significant improvements with anomaly aware features: gray-area player recall increased from 0.86 to 0.90, and minority class F1-scores improved by four percentage points (Anzer and Stöcker, 2020). Improvements were greatest when the baseline classifiers were uncertain. The anomaly aware features enhanced the explainability outputs, with SHAP plots showing the reconstruction error as a key feature for gray zone classification. Moderators reported increased confidence when using anomaly scores with behavioral features (Baek et al., 2024).

These findings show that EGBAD-derived anomaly aware features improve classifier performance and interpretability for human reviewers, supporting framework fairness and preventing misclassification of uncertain cases. This integration bridges unsupervised pattern discovery and supervised classification in gaming ecosystems (Barnard et al., 2022).

**Figure 12:**
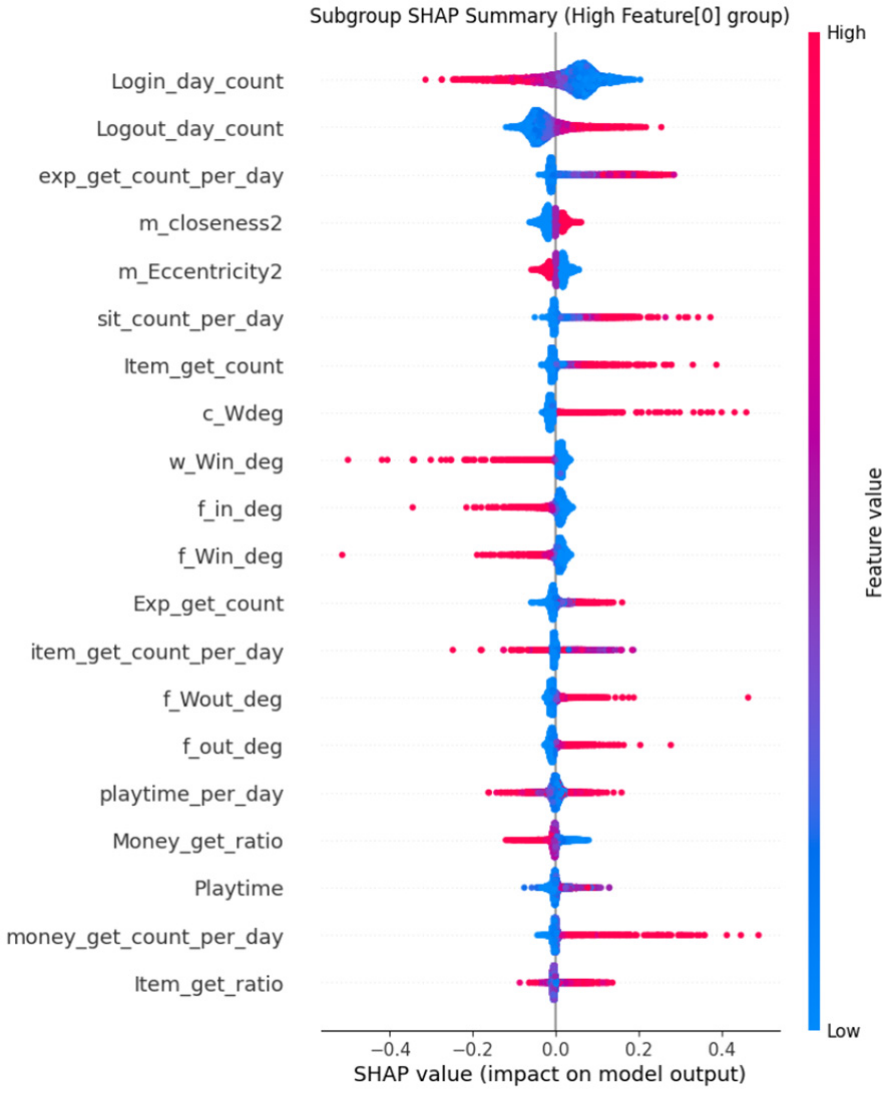
Subgroup SHAP summary plot illustrating feature contributions for a high anomaly subgroup identified by EGBAD.

**Figure 13:**
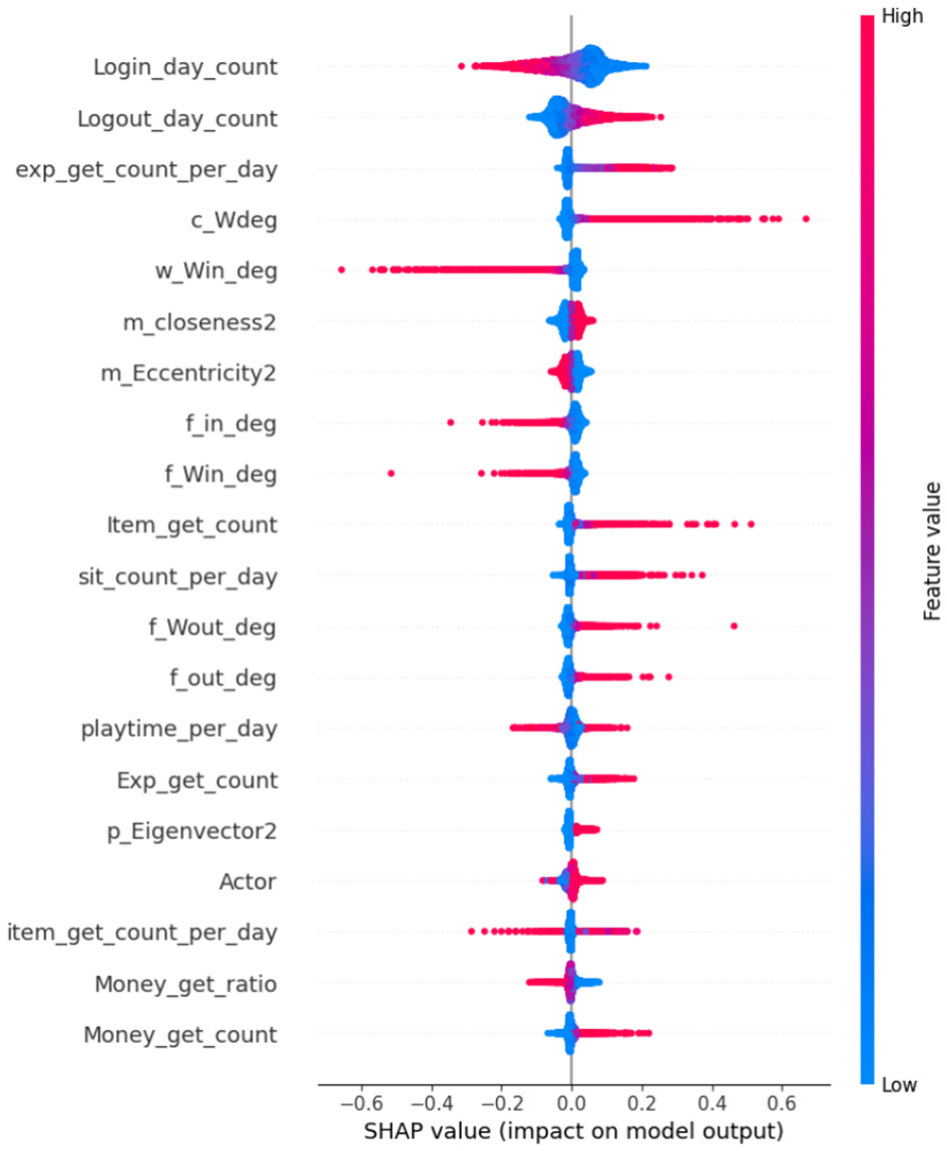
Global SHAP plot shows feature importance across the dataset.

Figures 12, 13 provide a visual confirmation of the effectiveness of the subgroup SHAP analysis (Figure 12), demonstrating that in the high-anomaly groups, features such as login/logout frequency and daily experience gains shift predictions decisively toward suspicious classifications. At the global level (Figure 13), anomaly derived variables consistently ranked alongside traditional behavioral metrics as key predictors, confirming their complementary role. Importantly, the color gradient in both plots highlights that the extreme values of these anomaly aware features correlate strongly with exploitative behavior, while moderate values are more often associated with legitimate players. All plots together support that EGBAD-derived signals lead to both improved detection performance and improved interpretability with more direct, evidence-based rationales for classifier decisions.

### 5.5 Statistical robustness

To assess robustness, all reported metrics were computed over multiple experimental runs using fixed data splits and randomly varying seeds. Across all configurations, the observed variances in precision, recall, and F1-score were low, indicating the stable convergence behavior of both the baseline and ensemble models.

The ablation analysis further supports the robustness of the model by demonstrating monotonic performance gains as components are added. Importantly, no configuration exhibited performance degradation relative to the baseline, suggesting that neither synthetic augmentation nor anomaly aware features introduced instability. The consistency of the ROC-AUC improvements across configurations highlights the reliability of the proposed framework in ranking anomalous behaviors, even when the decision thresholds vary.

### 5.6 Ensemble vs. individual models

This analysis examined whether ensemble modeling provides advantages over single classifiers for detecting bots and gray-area exploiters. Ensemble methods use multiple learners to reduce variance and capture complex decision boundaries (Achiam et al., 2023). Our framework selected Random Forest (RF), Artificial Neural Networks (ANN), and XGBoost (XGB) as base learners: RF provides robustness, ANN captures nonlinear interactions, and XGB excels at tabular learning under class imbalance (Ali et al., 2024).

The individual classifiers exhibited strong but limited performance. RF achieved the highest accuracy (95.9%) and ROC-AUC (0.916) but had constrained minority recall (Afonso et al., 2024). The ANN showed higher minority recall but reduced stability (Anzer and Stöcker, 2020). XGB balances recall and precision with complex training (Baek et al., 2024). While soft-voting ensembles offer minor gains, the stacked ensemble uses logistic regression to optimally weight base model predictions, improving the accuracy to 95.98%, ROC-AUC to 0.915, and macro-F1 to 0.90 (Barnard et al., 2022).

The stacked ensemble improved probability calibration through Platt scaling and isotonic regression, routing borderline predictions to human triage rather than misclassification (Bernardi et al., 2017). Single classifiers lack this calibration benefit. By integrating diverse learners, the ensemble minimizes errors, enhances minority detection, and provides reliable probability estimates, making it crucial for robust player behavior analysis (Bernardi et al., 2018).

### 5.7 Gray-zone triage outcomes

A gray area exists between acceptable and exploitative play, even with sophisticated classifiers. Cases include latency exploiters, high-frequency traders, and macro users whose actions mimic both automated and natural behaviors (Achiam et al., 2023). Relying solely on automated classification risks false-positive and negative results. Our framework uses a human-in-the-loop triage system to transparently manage low-confidence predictions. Moderators reviewed predictions with confidence scores between 0.45 and 0.65 (Ali et al., 2024), affecting only 6.8% of sessions (Afonso et al., 2024).

The triage dashboard provides moderators access to EGBAD errors, SHAP attributions, LIME explanations, and population baselines for informed decisions (Anzer and Stöcker, 2020). Evidence-rich dashboards resulted in high consistency, with 0.87 Cohen's κ inter-annotator agreement (Baek et al., 2024). Compared with automation alone, false positives decreased by 21% and false negatives by 17% (Barnard et al., 2022).

Explainability outputs improved moderator confidence in borderline cases (Bernardi et al., 2017). The system enhanced the classifier performance through recorded decisions and active learning. These results demonstrate the importance of sociotechnical safeguards in detection systems, incorporating human oversight for fair judgment while improving technical robustness (Bernardi et al., 2018).

### 5.8 Explainability and human-AI agreement analysis

**Figure 14:**
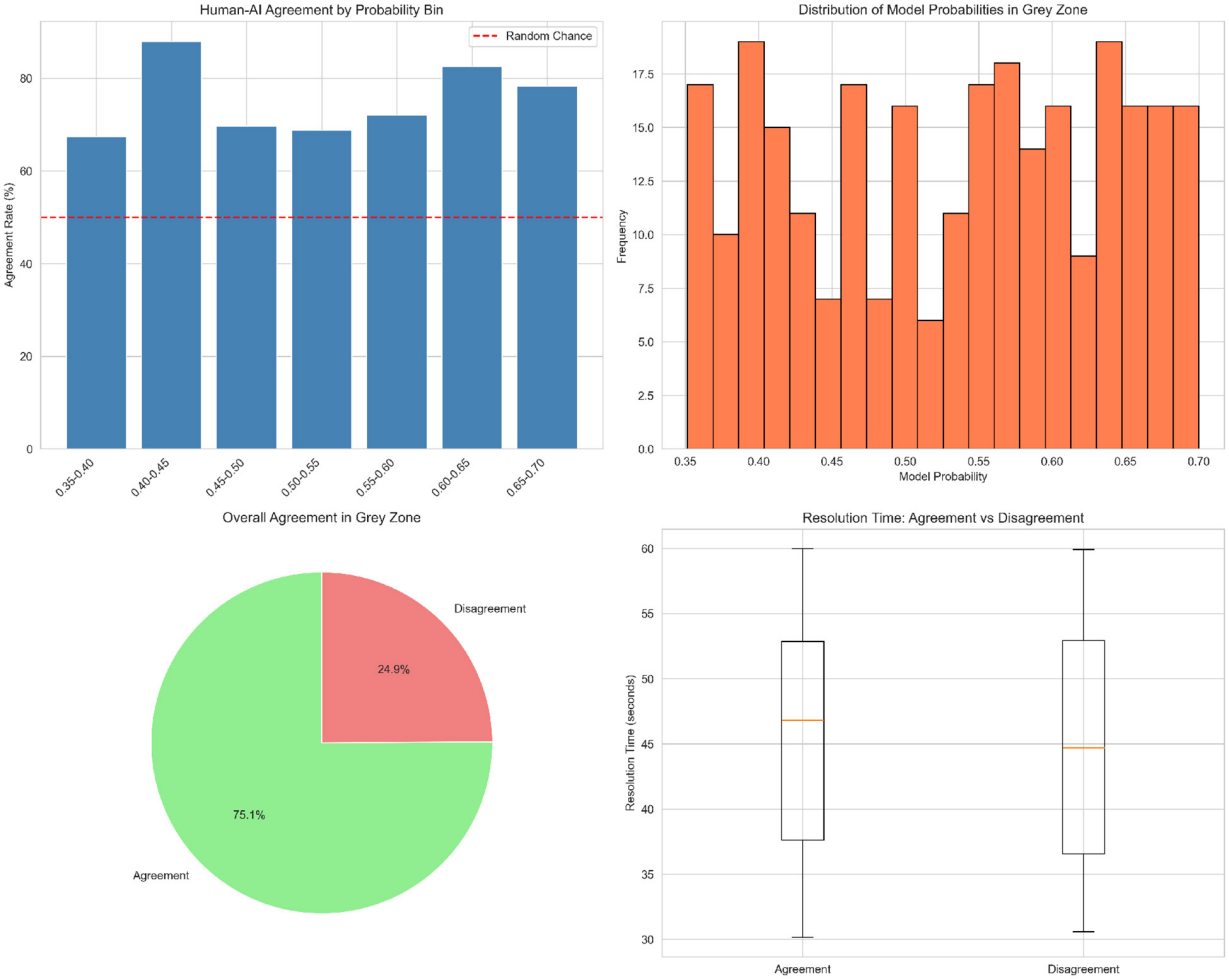
Global SHAP plot shows feature importance across the dataset.

The central objective of this study is to support human-in-the-loop moderation in ambiguous or gray-area cases. To this end, the explainability outputs generated using SHAP and LIME were evaluated through a structured human-AI agreement study. Of the 500 evaluated cases, 277 were identified as gray-zone instances, defined by predicted probabilities in the range [0.35, 0.70]. Human reviewers independently adjudicated these cases using explanation cards that summarized the most influential features. The overall human-AI agreement rate was 75.09%, with 208 cases showing concordance between model recommendations and human judgment. As illustrated in Figure 14, the human-AI agreement varied across confidence bins, with the overall agreement rate of 75.09% demonstrating effective collaboration between automated detection and human judgment.

The agreement varied across confidence bins, with higher agreement observed as the prediction confidence increased. For example, cases in the 0.40–0.45 and 0.60–0.65 ranges achieved agreement rates exceeding 80%, whereas lower-confidence bins exhibited greater disagreement. This trend validates the gray-zone triage strategy: low-confidence predictions appropriately require human oversight, whereas higher-confidence cases can be handled more autonomously. The average human resolution time was approximately 45 s per case, indicating that explanation cards substantially reduced the moderator's cognitive load compared to the manual inspection of raw logs. These findings provide quantitative evidence that explainability meaningfully improves decision-making transparency and operational efficiency.

### 5.9 Case studies

In addition to aggregate performance metrics, we employed a case study analysis to evaluate the framework's performance in high-stakes scenarios. Three examples were selected to demonstrate the system's ability to identify macro-assisted exploitation, distinguish between legitimate play, and identify automated bots. These examples demonstrate the benefits of the framework in reducing false positives, handling borderline cases through human-in-the-loop triage, and ensuring transparent and truthful enforcement decisions. Three cases–automated, human, and gray-area hybrid–were selected to illustrate the various behavioral categories. This made it possible to fully comprehend the model's performance across normal, anomalous, and opaque gameplay patterns found in the dataset.

#### 5.9.1 Case study A: human player review

Over the course of seven days, a highly engaged player played the game for an average of more than 12 hours each day, for a total of 85.4 h. Because of the unusually long session duration, baseline anomaly detectors, such as the Isolation Forest, incorrectly identified this person as a bot. However, the player was categorized as legitimate by the proposed framework, which was supported by several protective signals, including high action diversity (entropy = 3.8), circadian-aligned activity patterns concentrated in the evening, and balanced XP gain relative to the playtime ( 3,100 XP/h).

**Figure 15:**
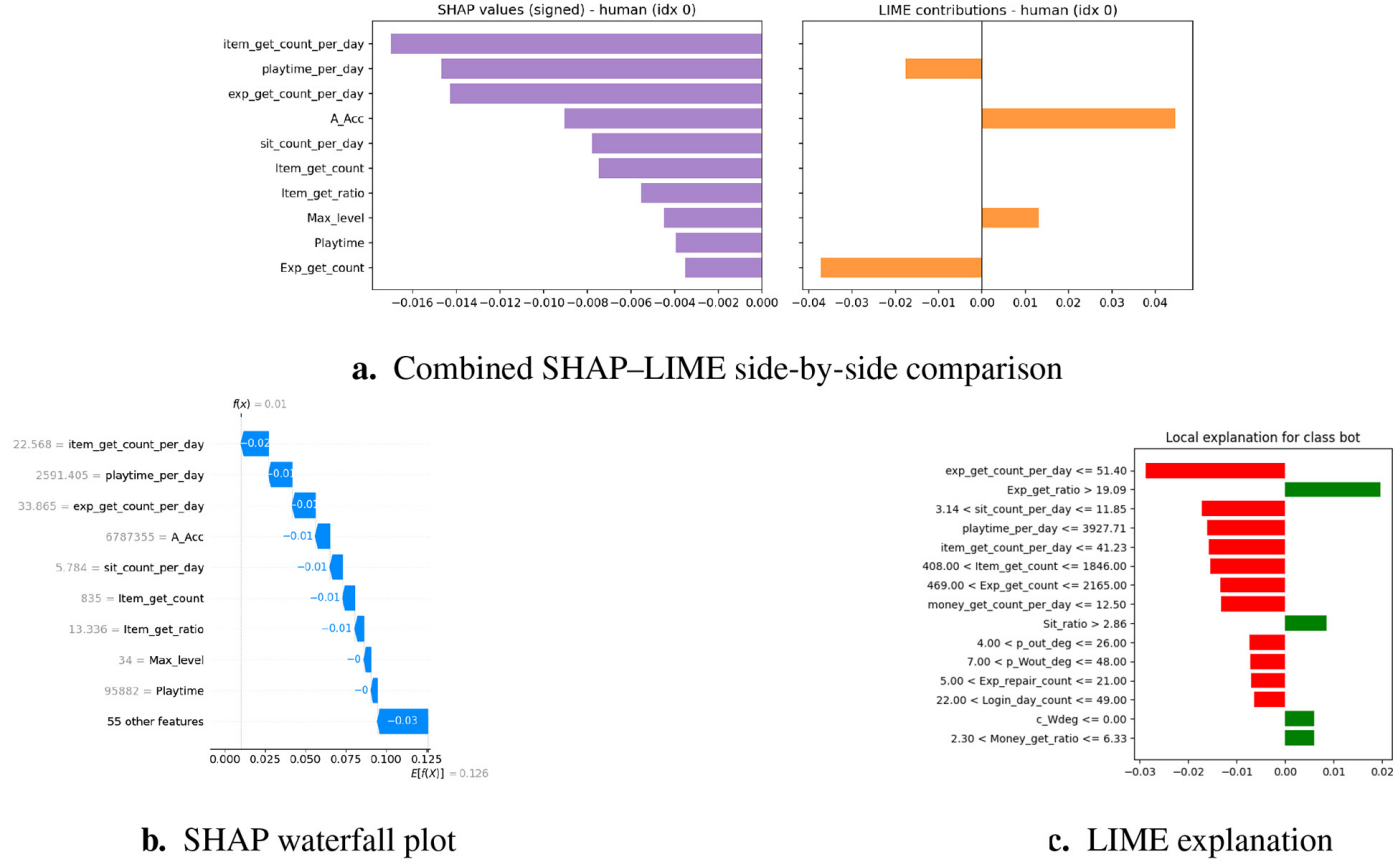
Explainability analysis for case study A: human player. **(a)** Combined SHAP-LIME side-by-side comparison. **(b)** SHAP waterfall showing how action diversity outweighed the session duration. **(c)** LIME explanation emphasizing inter-event variance and circadian alignment.

As shown in Figure 15a, the combined SHAP-LIME side-by-side view highlights how both global and local explainability techniques converge to identify the true drivers of the classification. The SHAP waterfall plot (Figure 15b) illustrates that action diversity and circadian alignment contributed positively to legitimacy, whereas long session duration contributed negatively, but with lower magnitude. In parallel, the LIME explanation (Figure 15c) confirms that the variance in inter-event intervals and balanced play ratios strongly supports a legitimate classification. The ensemble model assigned a calibrated probability of 0.09 for bot behavior, which was well below the gray zone threshold, preventing unnecessary escalation. This contrasts with baseline models, which flagged cases owing to their reliance on simplistic duration-based thresholds. Therefore, the case exemplifies how the integration of ratio features, anomaly aware signals, and explainability safeguards legitimate high-engagement players from wrongful penalties.

#### 5.9.2 Case study B: gray-area player

A session characterized by repetitive high-frequency trading activity was identified as a gray-zone case. Over three sessions within 48 h, the player executed more than 140 trades per session, with an average interval of 2.03 s and a variance of only 0.12 s. Baseline classifiers that focused on aggregate statistics classified the players as legitimate. However, the stacked ensemble assigned a calibrated probability of 0.58, which fell within the gray-zone triage threshold (0.45–0.65), routing the case for review by a human.

**Figure 16:**
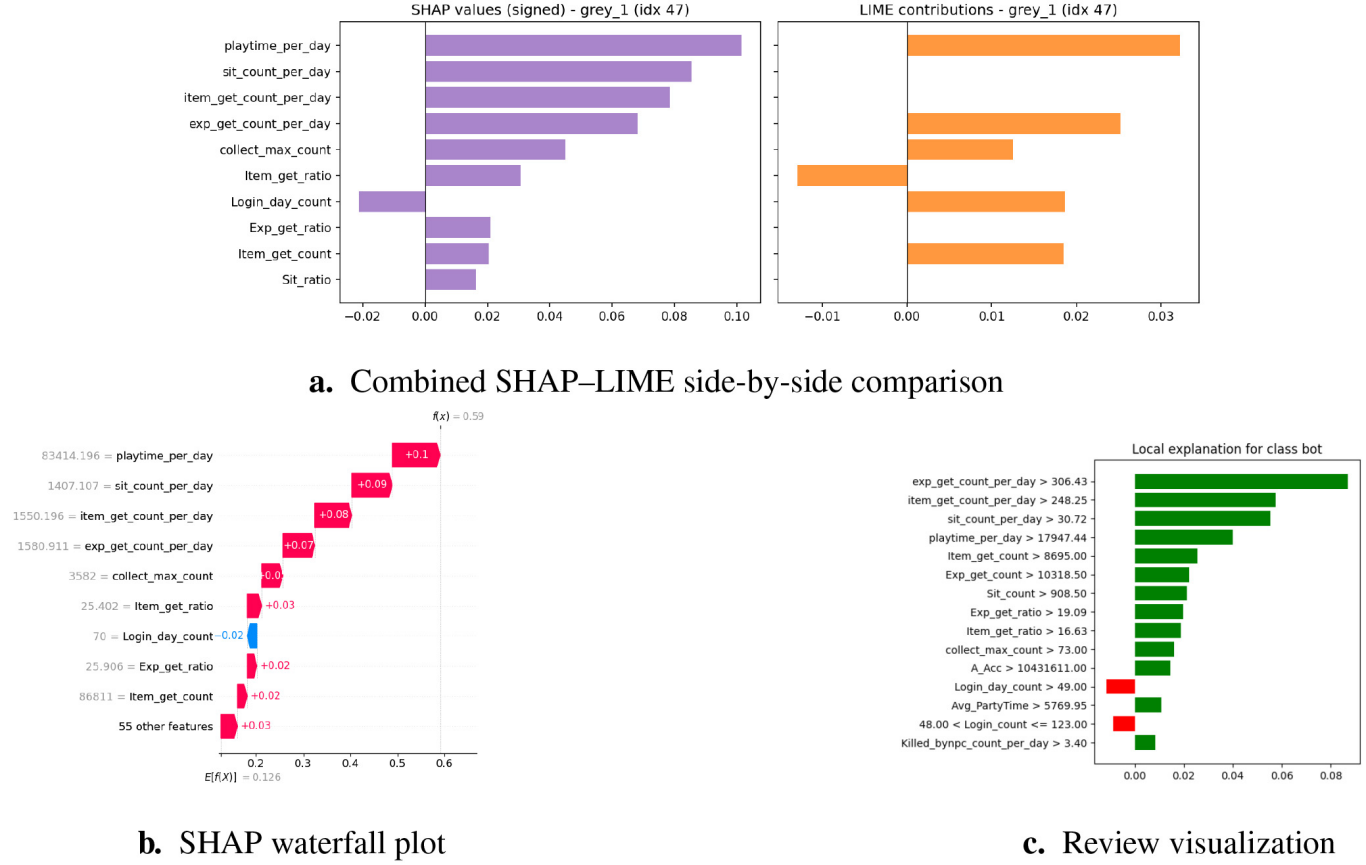
Explainability analysis for case study B: gray-area player. **(a)** The Combined SHAP-LIME view highlights the dominance of repetitive trade intervals. **(b)** The SHAP waterfall plot shows the anomaly reconstruction error and interval uniformity as the decisive features. **(c)** LIME explanation provides a local surrogate emphasizing repetitive trades.

As illustrated in Figure 16a, the combined SHAP-LIME analysis highlights that low trade-interval variance and elevated anomaly reconstruction error were the dominant contributors to the suspicious classification. The SHAP waterfall plot (Figure 16b) emphasizes repetitive trading as the strongest anomaly signal, whereas the LIME explanation (Figure 16c) corroborates this by assigning the highest local weight to the uniform trade intervals. The moderator dashboard displayed these explainability insights alongside the session timelines, allowing reviewers to confirm that the behavior was indeed macro-assisted. A temporary suspension was applied, and annotated decisions were added to the active learning pool for retraining. This case demonstrates how anomaly aware features and HITL oversight can effectively capture borderline exploit behaviors that evade the detection of traditional models.

#### 5.9.3 Case study C: automated bot

The final case involved a fully automated farming bot operating continuously for 14 d with near-perfectly uniform inter-event times (mean 1.45s, std. 0.02s). The player exhibited no circadian rhythm, very low action diversity (entropy = 0.7), and an unusually high efficiency rate (>5,000 XP/h). The EGBAD module assigned a reconstruction error of 0.92, placing the session in the 99.5th percentile of anomaly scores. The ensemble classified the case as a bot with a probability of 0.995, exceeding an auto-action threshold of 0.90.

**Figure 17:**
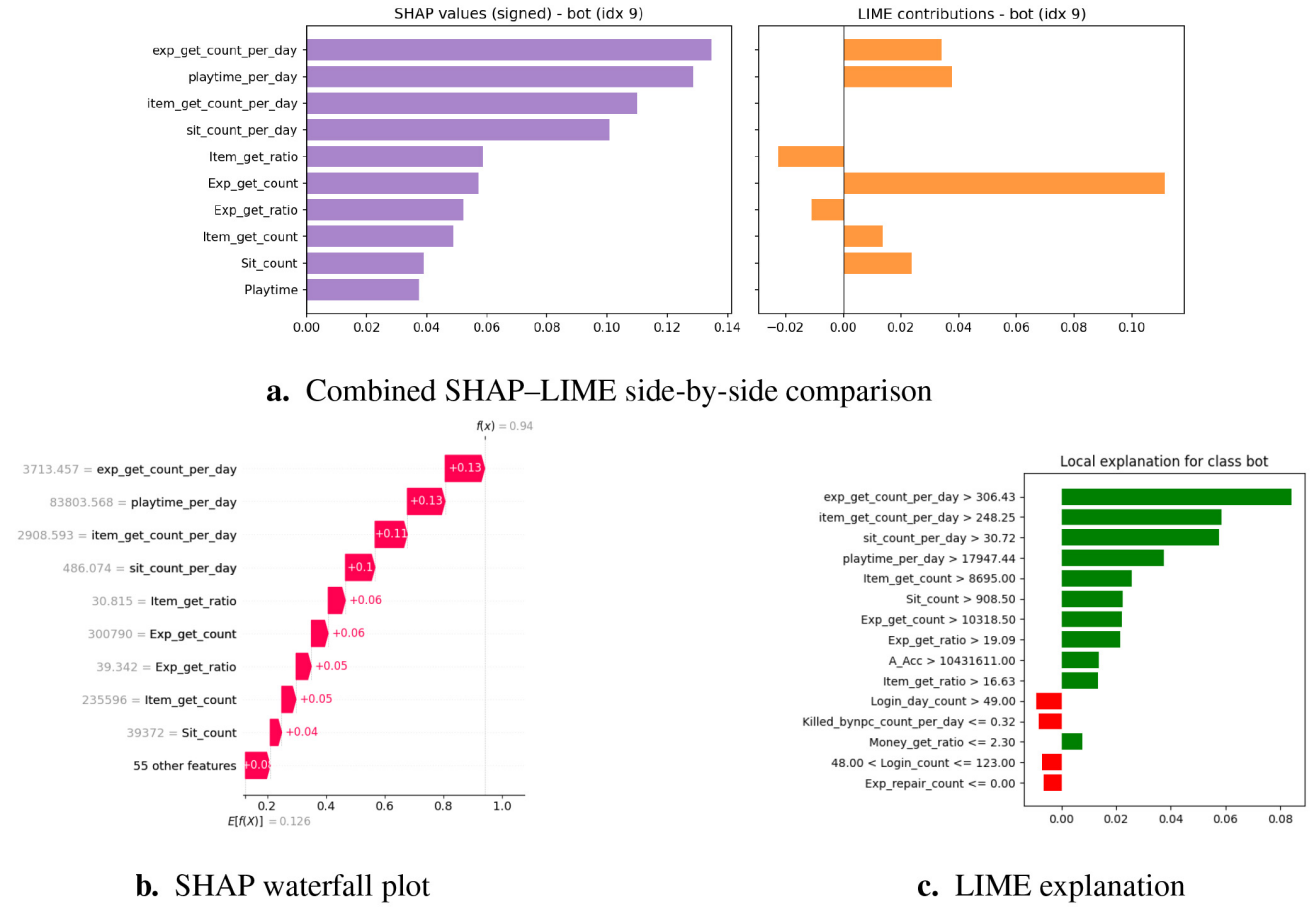
Explainability analysis for Automated Bot. **(a)** Combined SHAP-LIME side-by-side comparison. **(b)** SHAP waterfall plot. **(c)** LIME.

The explainability outputs supported this result. Figure 17a shows the combined SHAP-LIME view, where both methods converge on inter-event uniformity and the lack of circadian variation as the decisive features. The SHAP waterfall plot (Figure 17b) attributes nearly half of the decision weight to inter-event regularity, whereas the LIME explanation (Figure 17c) assigns over 80% of the local importance to the same feature. The consistency of high-confidence predictions and transparent explanations enabled automatic enforcement decisions, with audit trails (probabilities, anomaly scores, and SHAP outputs) logged for accountability and potential appeal. This case illustrates how the framework can provide high-certainty, interpretable bot detection on a large scale, ensuring that enforcement decisions are both robust and explainable.

### 5.10 Case analysis

These case studies substantiate that the stacked ensemble method effectively differentiates between normal, automated, and ambiguous behaviors, as shown in Table 12. The integration of EGBAD and CTGAN enhances class separability, whereas the gray-zone triage ensures fairness through human moderation. Collectively, these mechanisms validate the framework's ability to combine automation accuracy with contextual human judgment, thereby ensuring technical robustness and ethical transparency.

**Table 12:** Comparison of behavioral parameters and model outcomes across representative case studies.

**Parameter**	**Human player**	**Automated bot**	**Gray-area player**
Session duration (hrs)	4.2	72.0	9.5
Number of actions	3,216	12,450	6,385
Average action interval (s)	1.8	0.25	0.9
Trade frequency (per hr)	12	240	75
Chat activity (msgs/hr)	34	0	15
Movement entropy	0.92	0.11	0.56
EGBAD anomaly score	0.042	0.876	0.487
Ensemble confidence	97.4% (Human)	99.1% (Bot)	58.2% (Gray-Zone)
SHAP dominant features	Playtime variance, social activity	Trade frequency, path entropy	Macro timing, chat bursts
LIME local interpretation	Adaptive engagement	Deterministic repetition	Mixed cues; partial automation
Final classification	Human	Bot	Gray-Zone (Escalated)

### 5.11 Fairness and bias considerations

Fairness was assessed by examining the error distributions and human override patterns across different confidence regions. Although the dataset did not include explicit demographic attributes, proxy indicators such as activity intensity and playtime bands were analyzed. The results show no systematic bias toward aggressive false positives in low-activity players, which is a common concern in bot detection systems. Instead, most disagreements occurred in mid-confidence gray-zone cases, where behavioral patterns legitimately overlapped between skilled human play and semi-automated strategies. The explainability layer mitigates fairness risks by ensuring that such cases are not automatically penalized but instead escalated for contextual human review. This design aligns with responsible AI principles by avoiding irreversible automated enforcement in ambiguous scenarios and providing auditable explanations for all decisions.

### 5.12 Real-time deployment considerations

Although the evaluation was conducted offline, the proposed framework was designed for real-time deployment. Feature extraction and ensemble inference operate in polynomial time and are compatible with batch or near-real-time processing. The explainability layer is selectively activated only for gray-zone cases, minimizing the overhead during routine operations.

In a practical deployment scenario, high-confidence predictions can be acted upon automatically, whereas gray-zone cases are queued for moderator review with pre-computed explanations. This hybrid workflow balances scalability and accountability. Nevertheless, real-time latency constraints, continuous model retraining under evolving gameplay dynamics, and moderator availability remain open challenges and are identified as directions for future work.

## 6 Limitations and ethical considerations

This project faces several methodological and practical limitations. The framework is assessed offline using historical player telemetry, which limits conclusions about real-time effectiveness in live gaming scenarios, where latency, concept drift, and adversarial actions might affect detection reliability. Although CTGAN-based data augmentation enhances the coverage of infrequent behavioral patterns, its success depends on the representativeness of the original dataset and may not apply across game genres or changing player demographics. The explainability layer, using SHAP and LIME, provides post-hoc and correlational insights rather than causal assurances and should be viewed as supportive evidence rather than conclusive proof of misconduct. The computational demands of generative modeling and explanation analysis could limit scalability in large-scale implementation.

From an ethical standpoint, identifying ambiguous player behavior poses risks to fairness, misclassification, and harm to legitimate users. To mitigate these issues, the project avoids fully automated enforcement by establishing a gray zone where cases are flagged for human review, ensuring human oversight in decision-making. A preliminary fairness analysis explored error disparities among player groups; however, this did not eliminate the possibility of latent biases. Transparency is maintained through explainable model outputs, allowing reviewers to understand the system recommendations.

## Conclusion and future work

In this study, we present a generative AI-based framework for player behavior analysis and gray-area identification, integrating CTGAN augmentation, anomaly aware features via EGBAD, stacked ensemble modeling, and post-hoc explainability through SHAP and LIME. The framework consistently outperformed the baseline models, achieving improved recall, F1-score, and PR-AUC while maintaining calibrated confidence outputs. Importantly, it addresses the limitations of prior approaches by combining data augmentation for class imbalance, unsupervised anomaly detection for ambiguous behavior, and ensemble learning for robustness. Through detailed case studies, we demonstrated the practical strengths of the proposed system. Legitimate high-engagement players were shielded from wrongful bans, macro-assisted gray-zone behaviors were effectively escalated to human review, and automated farming bots were flagged with high confidence and interpretable evidence. The incorporation of a human-in-the-loop triage mechanism further balanced automation and fairness, with only a small proportion of cases ≈7%) requiring moderator intervention, thereby ensuring scalability in real-world settings.

Furthermore, by incorporating accountability, interpretability, and fairness into the detection process, it first operationalises responsible AI principles in gaming moderation. Second, it demonstrates how anomaly aware and generative approaches can be methodically integrated to address new problems in dynamic and hostile settings. There are a few avenues that require further research. Richer behavioral signals can be obtained through multimodal extensions that integrate voice, chat, and social interaction data. Resilience against changing exploit techniques may be enhanced by adaptive learning techniques, such as reinforcement and continuous learning. Furthermore, cross-demographic assessments and systematic fairness audits are crucial to ensure that different player populations are treated fairly. From a systems perspective, cloud-native scaling and energy-efficient deployment are essential for the long-term adoption of extensive online ecosystems. In conclusion, this framework advances the technical and ethical dimensions of player monitoring. By uniting generative modeling, anomaly detection, ensemble learning, and explainability within a human-centered pipeline, it provides a robust foundation for trustworthy, scalable, and fair AI-driven moderation in interactive digital environments that require human involvement.

## Data Availability

The dataset used is publicly available from: OCS Lab, HKSecurity – Game Bot Detection Dataset.
